# A novel antisense long noncoding RNA within the *IGF1R* gene locus is imprinted in hematopoietic malignancies

**DOI:** 10.1093/nar/gku549

**Published:** 2014-08-04

**Authors:** Jingnan Sun, Wei Li, Yunpeng Sun, Dehai Yu, Xue Wen, Hong Wang, Jiuwei Cui, Guanjun Wang, Andrew R. Hoffman, Ji-Fan Hu

**Affiliations:** 1 Stem Cell and Cancer Center, First Affiliated Hospital, Jilin University, Changchun, Jilin 130061, PR China; 2 Stanford University Medical School, VA Palo Alto Health Care System, Palo Alto, CA 94304, USA

## Abstract

Dysregulation of the insulin-like growth factor type I receptor (*IGF1R*) has been implicated in the progression and therapeutic resistance of malignancies. In acute myeloid leukemia (AML) cells, *IGF1R* is one of the most abundantly phosphorylated receptor tyrosine kinases, promoting cell growth through the PI3K/Akt signaling pathway. However, little is known regarding the molecular mechanisms underlying *IGF1R* gene dysregulation in cancer. We discovered a novel intragenic long noncoding RNA (lncRNA) within the *IGF1R* locus, named *IRAIN*, which is transcribed in an antisense direction from an intronic promoter. The *IRAIN* lncRNA was expressed exclusively from the paternal allele, with the maternal counterpart being silenced. Using both reverse transcription-associated trap and chromatin conformation capture assays, we demonstrate that this lncRNA interacts with chromatin DNA and is involved in the formation of an intrachromosomal enhancer/promoter loop. Knockdown of *IRAIN* lncRNA with shRNA abolishes this intrachromosomal interaction. In addition, *IRAIN* was downregulated both in leukemia cell lines and in blood obtained from high-risk AML patients. These data identify *IRAIN* as a new imprinted lncRNA that is involved in long-range DNA interactions.

## INTRODUCTION

Dysregulation of the genes encoding members of the insulin-like growth factor axis, including the receptor *IGF1R* and the ligands *IGF1* and *IGF2*, can contribute to the progression and metastasis of human cancers (1–5). In cells from patients with acute myeloid leukemia (AML), IGF-I and IGF-II promote cell growth and survival via IGF1R receptor-mediated activation of the PI3K/Akt signaling pathway (6–8). In clinical samples, AML blasts contain high concentrations of IGF-I/II, resulting in a growth and survival advantage and increasing the survival of leukemia cells through autocrine and paracrine loops (9). *IGF1R* is one of the most abundantly phosphorylated receptor tyrosine kinases in leukemia cells (10–12), and phosphorylation is increased in leukemia cells with Ara-C resistance (13,14) The IGF1R inhibitor BMS-536924 substantially inhibited growth and proliferation of both mouse and human leukemia cells *in vitro* (15).

Numerous clinical cancer trials have been performed that target *IGF1R* (16–20), including those with drugs that inhibit the IGFIR tyrosine kinase using monoclonal antibodies and small molecules (21). However, little is known regarding the mechanism by which *IGFIR* becomes dysregulated in tumors. Using a novel R3C (**R**NA-guided **C**hromatin **C**onformation **C**apture) method recently developed in our lab (Supplementary Figure S1) (22), we demonstrate the presence of a novel long noncoding RNA (lncRNA) originating from the *IGF1R* promoter. lncRNAs have been implicated in a number of regulatory functions in eukaryotic genomes (23–25), including the epigenetic regulation in *cis* and in *trans* of a cluster of genes within large chromosomal domains (26–30). In this communication, we characterize the allelic expression of *IRAIN* lncRNA and its role in the formation of interchromosomal interactions in normal and tumor cells.

## MATERIALS AND METHODS

### Cell lines

Leukemia cell lines used in this study, K562, KG-1, KG-1a, HL60 and TF1, were purchased from ATCC. Cells were grown in RP1640 Media, supplemented with 10% FBS, 100 U/ml penicillin and 100 μg/ml streptomycin.

### AML and peripheral blood cell samples

The protocol was approved by the Human Medical Ethical Review Committee from Jilin University First Hospital and informed consent was obtained from each AML patient and normal subject. Bone marrow samples were obtained from 34 AML patients at diagnosis and 10 healthy volunteers in Jilin University First Hospital (Supplementary Table S1) in Changchun City, China. AML patients were classified into high-risk and low-risk groups by cytogenetics and molecular abnormalities according to the NCCN guidelines (version 2.2013). The low-risk group (*n* = 18) was defined as patients with t(8;21) or RUNX1-RUNX1T1, inv(16), t(16;16) or CEBF-MYH11, normal karyotype with NPM1 mutation and without FLT3-ITD mutation, and normal karyotype with isolated biallelic CEBPA mutation (normal karyotype). The high-risk group (*n* = 16) included patients with inv(3), t(3;3) or RPN1-EVI1, t(6;9) or DEK-NUP214, t(9;22) or BCR-ABL, t(v;11) (v;q23), MLL rearranged, −5 or del (5q), −7 or del (7q), complex karyotype, monosomal karyotype, normal karyotype with FLT3-ITD mutation (Supplementary Table S2).

Leukocyte fractions from AML samples and normal bone marrow specimens were isolated by Ficoll-Hypaque (Sigma, MO) centrifugation and then cryopreserved. After thawing, total RNA was extracted by RNeasy Kit (Qiagen, CA) for qPCR quantitation.

### Reverse transcription-PCR analysis

Total RNA was extracted from tissues by TRI-REAGENT (Sigma, CA), according to the manufacturer's guide, and cDNA was synthesized with RNA reverse transcriptase as previously described (31,32). Briefly, 1 μg of total RNA was used, and polymerase chain reaction (PCR) was carried out under liquid wax in a 6 μl reaction containing 2 μl of 3× Klen-T*aq* I Mix, 2 μl cDNA and 1 μl of each 2.5 μM primer. After incubation at 95°C for 2 min, *IRAIN* cDNA was amplified by 32 cycles of 95°C for 30 s, 65°C for 30 s of annealing and 72°C for 35 s of extension, and finally with extension at 72°C for 5 min. Amplified PCR products of the expected size were quantified by densitometric measurements and normalized to ‘β-actin’ values.

### Gene strand-specific RT-PCR

A strand-specific PCR (SSRT) assay was used to map the transcription of the *IRAIN* lncRNA. Total RNA was extracted from tissues by TRI-REAGENT (Sigma, CA), according to the manufacturer's guide, and cDNA was synthesized with reverse transcriptase using gene specific primers. Briefly, 400 ng total RNA was reverse transcribed with the *IRAIN* 5′- or 3′-primers instead of random hexamers. The RT reaction was performed with Maxima Reverse Transcriptase (Thermo Fisher Scientific, CA) at 60? for 50 min, followed by 85? for 5 min to inactivate the transcriptase. After 10-fold dilution, PCR was carried out under liquid wax in a 6 μl reaction containing 2 μl of 3 × Klen-T*aq* I Mix, 2 μl cDNA and 1 μl of each 2.5 μM downstream PCR primer set. After initial denaturing at 95°C for 2 min, *IRAIN* cDNA was amplified by 32 cycles at 95°C for 30 s, 65°C for 30 s of annealing and 72°C for 35 s of extension, followed by incubation at 72°C for 5 min. PCR products with the expected size were quantified by densitometric measurements and normalized to ‘β*-*actin’ values.

### Characterization of the *IRAIN* lncRNA by 5′ racing

The full length of *IRAIN* lncRNA was characterized by Marathon cDNA Amplification Kit (Clonthech, CA) (33). Total RNA was reverse transcribed into cDNA. The 5′- and 3′-ends were raced using two primers that cover a unique *Bam* H1 site in the middle of the *IRAIN* lncRNA: JH1095 (forward): GGCTCGCTGAAGGTCACAGC and JH986 (reverse): AGGCTGGGGCTCTTGTTTACCA. The 5′- and 3′-RACE products were cloned into pJet vector (Thermo Fisher Scientific, CA) and sequenced. The full-length *IRAIN* lncRNA was constructed by joining these two RACE products in a pCMV-MS-pEF1-coGFP/Puro lentiviral vector using two steps of Xba1-BamH1 and BamH1-EcoR1 cloning. The 5′- and 3′-end of the *IRAIN* lncRNA was determined by sequencing.

### Gene expression by real-time qPCR

After removing genomic DNA contamination with DNase I (Sigma), M-MLV Reverse Transcriptase (Invitrogen, CA) was used to synthesize cDNA (31,34). For qPCR, cDNA samples were amplified using CFX96^TM^ real-time system (BIO-RAD) by SYBR PrimeScript™ RT-PCR Kit (TaKaRa) (35). The mRNA expression level of *IRAIN* and *IGF1R* was quantitated by normalizing with β-actin (housekeeping gene) as previously described (34,36). PCR primers used for qPCR included (1) *IRAIN*: 5′-CGACACATGGTCCAATCACTGTT-3′ (forward) and 5′-AGACTCCCCTAGGACTGCCATCT-3′ (reverse); and (2) *IGF1R*: 5′-GAAGTCTGGCTCCGGAGGAGGGTC-3′ (forward) and 5′-ATGTGGAGGTAGCCCTCGATCAC-3′ (reverse); and β-actin: 5′-AGATCAAGATCATTGCTCCTCCTGA-3′ (forward) and 5′-ATACTCCTGCTTGCTGATCCACATC-3′.

### Northern analysis

Total RNA from breast cancer samples was separated by electrophoresis on a 1.5% (w/v) denaturing agarose gel, transferred to a Hybond-N nylon membrane (Amersham, UK) and cross-linked with UV light. The probe was prepared from the *IRAIN* clone DNA with ^32^P-dCTP labeling using Megaprime DNA Labelling Kit (Amersham, UK). The membrane was prehybridized in Rapid-hyb buffer (Amersham) for 30 min, followed by hybridization with the labeled probe at 65°C for 2 h. The membrane was exposed to X-ray film overnight and the image was scanned by a densitometer.

### Reverse transcription-associated trap assay

A ‘reverse transcription-associated trap’ (RAT) assay was developed for the measurement of RNA–DNA interactions (Supplementary Figure S2). Briefly, 1.0 × 10^7^ cells were cross-linked with 2% formaldehyde and lysed with cell lysis buffer (10 mM Tris [pH 8.0], 10 mM NaCl, 0.2% NP-40, 1× protease inhibitors). Nuclei were collected, suspended in 1× reverse transcription buffer in the presence of 0.3% sodium dodecyl sulfate (SDS) and incubated at 37°C for 1 h. Triton X-100 was then added to a final concentration of 1.8% to sequester the SDS. An aliquot of nuclei (3 × 10^6^) was used for gene strand-specific reverse transcription in the presence of biotin-dCTP. For the parallel controls, we established three groups (5′-prime RT, 3′-prime RT and non-prime RT group). After 50 min of reverse transcription of *IRAIN* lncRNA with Maxima Reverse Transcriptase (Thermo Fisher Scientific, CA) at 60°C, the reaction was stopped by heating at 85°C for 5 min. The biotinylated-cDNA/chromatin DNA complex was diluted with 200 μl 1× NEB EcoRI buffer and digested at 37°C for 2.5 h with rotation by 600 μl EcoRI (New England BioLabs, CA). Biotin-streptavidin magic beads (Invitrogen, CA) were used to pull down the biotinylated-cDNA/DNA complex. After washing, the pull-down sample was treated with 10 mg/ml proteinase K at 65°C overnight to reverse the cross-links. Following incubation with 0.4 μg/ml RNase A for 30 min at 37°C, DNA was extracted and used for PCR amplification of the genomic DNA that interacts with the lncRNA.

### Chromosome conformation capture

The chromosome conformation capture (3C) assay was performed as previously described (37). Briefly, 1.0 × 10^7^ cells were cross-linked with 2% formaldehyde and lysed with cell lysis buffer (10 mM Tris [pH 8.0], 10 mM NaCl, 0.2% NP-40, protease inhibitors). Nuclei were collected, suspended in 1× restriction enzyme buffer in the presence of 0.3% SDS and incubated at 37°C for 1 h. Triton X-100 was then added to a final concentration of 1.8% to sequester the SDS. An aliquot of nuclei (2 × 10^6^) was digested with 800 U of restriction enzyme Hind III at 37°C overnight. After stopping the reaction by adding 1.6% SDS and incubating the mixture at 65°C for 20 min, chromatin DNA was diluted with NEB ligation reaction buffer, and 2 μg DNA was ligated with 4000 U of T4 DNA ligase (New England BioLabs, CA) at 16°C for 4 h (final DNA concentration, 2.5 μg/ml). After treatment with 10 mg/ml proteinase K at 65°C overnight to reverse cross-links and with 0.4 μg/ml RNase A for 30 min at 37°C, DNA was extracted with phenol-chloroform, ethanol precipitated and used for PCR amplification of the ligated DNA products.

### Chromatin immunoprecipitation

Chromatin immunoprecipitation (ChIP) assays were performed as described previously (38,39). Briefly, 5 million cells were fixed with 1% formaldehyde and sonicated for 180 s (10 s on and 10 s off) on ice with a Branson sonicator with a 2-mm microtip at 40% output control and 90% duty cycle settings. The sonicated chromatin (1 ml) was clarified by centrifugation, aliquoted and snap-frozen in liquid nitrogen. To perform ChIP, sonicated chromatin (150 μl) was diluted 10-fold and purified with specific antiserum (2–5 μl) and protein G-agarose (60 μl). ChIP antibodies were obtained from Abcam (Cambridge, MA), including anti-trimethyl-H3-K4 (#ab8580), trimethyl-H3-K9 (#ab8898) and trimethyl-H3-K27 (#ab24684). DNA that was released from the bound chromatin after cross-linking reversal and proteinase K treatment was precipitated and diluted in 100 μl of low-TE buffer (1 mM Tris, 0.1 mM EDTA). PCR reactions were performed under liquid wax in a reaction containing 1 μl ChIP (or input) DNA, 0.5 μM appropriate primer pairs, 50 μM deoxynucleotide triphosphate and 0.2 U Klen-T*aq* I (Ab Peptides, MO). Standard PCR conditions were 95°C for 2 min, followed by 32 cycles of 95°C for 15 s, 65°C for 30 s of annealing and 72°C for 30 s of extension. The PCR products were separated on a 5% polyacrylamide–urea gel and quantified by a Phosphorimager (Molecular Dynamics, CA).

### Examination of genomic imprinting

Total RNA extraction and cDNA synthesis were performed as previously described (31). Allelic expression of *IRAIN* and *IGF1R* was examined by PCR in cDNA samples using primers specific for polymorphic restriction enzymes. Allelic expression of *IRAIN* was assessed by polymorphic restriction enzymes *Alu* II and *Sac* II, and of IGF1R by *Rsa* II and *Pvu* II. In some cases, DNA sequencing of genomic DNA and cDNA PCR products was used to determine allelic expression of *IRAIN*. PCR primers used to assess allelic expression are listed in Supplementary Table S3.

### α-Amanitin treatment of KG-1 cells

KG-1 cells were cultured in RPMI1640 medium to 70–80% confluence. As previously described (40,41), cells were treated with α-amanitin (75 μg/ml) (Sigma, MO) for 5 h to block nascent mRNA synthesis. After depletion of pre-mRNA, cells were collected by centrifuge at 1000 rpm for 5 min and saved for RAT assay.

### Knockdown of *IRAIN* lncRNA by shRNA

Two *IRAIN* shRNAs (shIRAIN-1 and shIRAIN-2) were synthesized by GeneChem (Shanghai, China) and were transfected into MB-MDA231 cells. Forty-eight hours after transfection, cells were selected by puromycin and were collected for both *IRAIN* lncRNA quantitation using qPCR and intrachromosomal interaction using 3C assay.

### Lentiviral expression of *IRAIN* lncRNA

The full-length 5.4 kb *IRAIN* lncRNA was amplified with PCR primers containing the Xba1 and EcoRV restriction sites. The PCR products were gel-purified, cut by restriction enzymes and ligated into the pCMV-MS-EF1-copGFP/Puro vector constructed in the lab. The *IRAIN* lncRNA clone was confirmed by sequencing and then packaged in 293SF-PacLV packing cells (42) using the method described in our lab (22,35). After transduction, cell clones were selected by 1 μg/μl puromycin and used for cell migration assay (35).

### Statistical analysis

All experiments were performed in triplicate, and the data were expressed as mean ± SD. The data were analyzed with Student's *t*-test or by one-way analysis of variance, and results were considered statistically significant at *P* ≤ 0.05.

## RESULTS

### Identification of a novel antisense noncoding RNA in the IGF1R locus

**Figure 1.**
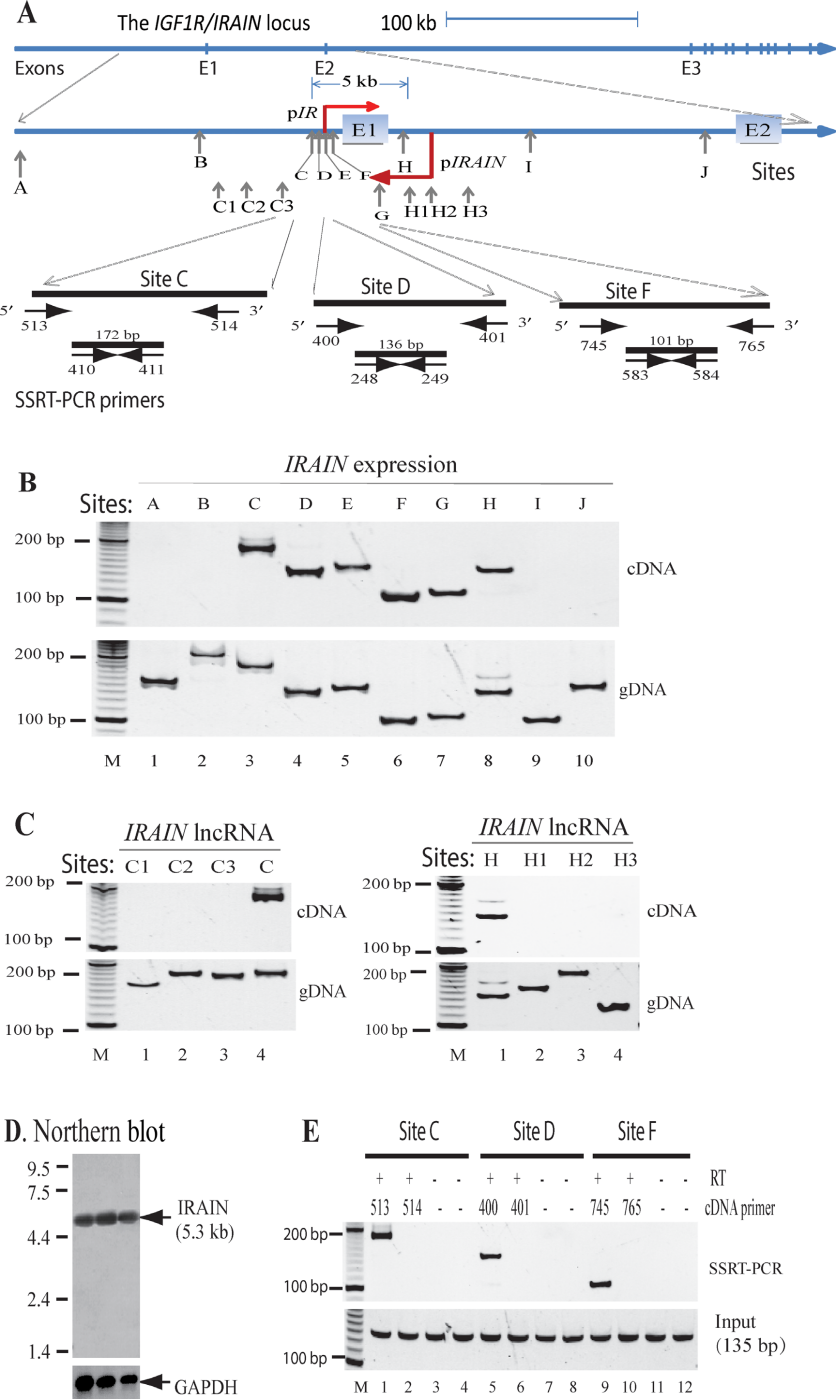
Characterization of *IRAIN* as an antisense lncNRA. (**A**) The diagram of the *IRAIN*/*IGF1R* locus. p*IRAIN*: *IRAIN* lncRNA promoter; p*IR*: *IGF1R* coding RNA promoter. Vertical arrows: the location of lncRNA PCR primers. (**B** and **C**) Mapping of the *IRAIN* lncRNA in K562 leukemia cells. gDNA: genomic DNA used as the control to test the efficiency of the PCR primers. M: 100 bp marker. (**D**) Northern blot of the *IRAIN* lncRNA in breast cancer tissues. Total RNA from three breast cancer tumors was separated on a 1.5% (w/v) denaturing agarose gel and was hybridized with the ^32^P-dCTP labeled *IRAIN* cDNA clone probe. GAPDH was used as the control. E: *IRAIN* lncRNA is an antisense lncRNA. Horizontal arrows: SSRT-PCR primers used to map the orientation of *IRAIN* lncRNA. The strand-specific cDNAs were synthesized using either the 5′- or the 3′-oligonucleotides at sites C, D and F. A pair of PCR primers located between two cDNA oligonucleotides was then used to determine the transcription orientation of the *IRAIN* lncRNA. M: 100 bp marker; input: total RNA collected before SSRT-PCR; RT: reverse transcriptase.

*IGFIR* is frequently overexpressed in both solid tumors and hematopoietic malignancies, participating in the regulation of cancer cell proliferation, survival, metabolism and metastasis. To address the mechanisms underlying the dysregulation of *IGF1R* in leukemia cells, we used a novel R3C method recently developed in our lab (Supplementary Figure S1) (22), and detected the presence of RNA molecules within the *IGF1R* promoter and intron 1, where the *IGF1R* mRNA is not transcribed. To characterize this novel RNA molecule, we designed a series of PCR primers covering the *IGF1R* promoter region and mapped its transcription in the *IGF1R* locus (Figure 1A). Using PCR, we detected the transcription of this noncoding RNA from the *IGF1R* promoter and intron 1 (Figure 1B, from C to H sites, lanes 3–8). No PCR products were detected further upstream (sites A, B; lanes 1–2) or the region near exon 2 (sites I, J; lanes 9–10).

The transcription of this new RNA was further mapped using sequential primers covering the upstream C1–C3 sites and the downstream H1–H3 sites (Figure 1A, middle panel). No PCR products were detected using PCR primers at these sites (Figure 1C), indicating that this noncoding RNA is located between the C3 and H1 sites covering all of the *IGF1R* promoter and exon 1. Using a 5′-racing approach, we characterized *IRAIN* as a 5366 bp noncoding RNA (Supplementary Figure S2). The presence of full-length *IRAIN* lncRNA was further confirmed by northern blotting in three breast cancer samples (Figure 1D).

We then used strand-specific RT-PCR (SSRT) to examine whether this RNA is transcribed in a sense or antisense direction. Total RNA was extracted from leukemia K562 cells. Strand-specific reverse transcription (SSRT) cDNA was synthesized by thermo-stable reverse transcriptase (Thermo Fisher Scientific, PA) utilizing a 5′-specific oligonucleotide or a 3′-specific oligonucleotide, respectively. After SSRT, a pair of PCR primers designed to detect regions downstream of the 5′- or 3′-oligonucleotide was used to amplify the strand-specific cDNA. With this assay, only the lncRNA that is complementary to the 5′- or 3′-oligonucleotide was reverse transcribed in the subsequent PCR using the downstream primers (Figure 1E, top panel).

Using this SSRT assay, we found that this noncoding RNA was detected only when cDNA was synthesized using 5′-oligonucleotides (#513, #400 and #744) (Figure 1E, lanes 1, 5 and 9). No PCR products were amplified when the 3′ oligonucleotides were used (#514, #401 and #764; lanes 2, 6 and 10) or in the RT-minus controls (lanes 3–4, 7–8 and 11–12). These data indicate that this noncoding RNA is transcribed in the antisense direction as compared with the *IGF1R* coding RNA. The transcription was initiated from a promoter located in the *IGF1R* intron 1. We therefore refer to the noncoding RNA as *IRAIN* (*IGF1R* antisense intragenic noncoding RNA).

### *IRAIN* lncRNA is imprinted in hematopoietic cells

**Figure 2.**
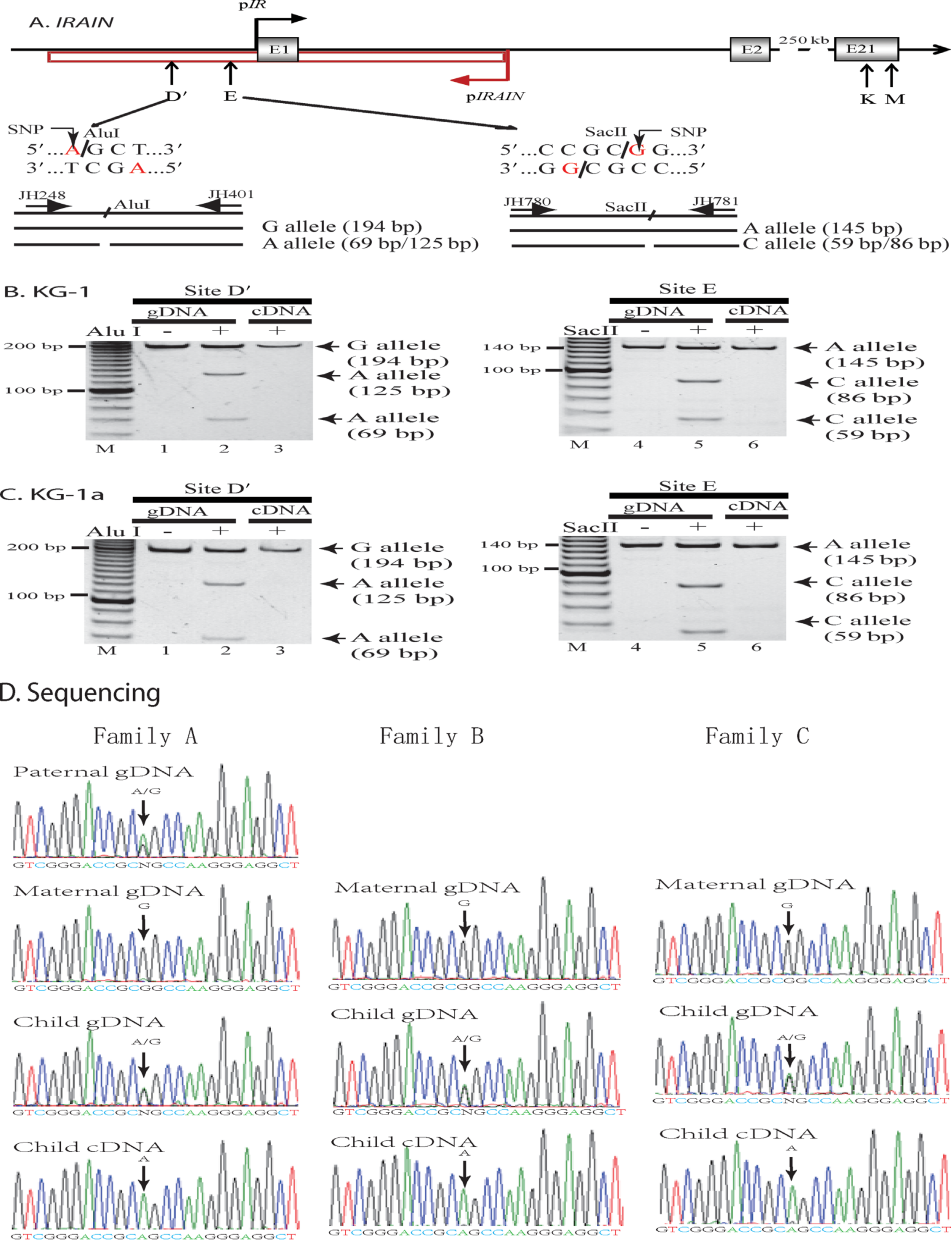
The *IRAIN* lncRNA is imprinted in hematopoietic cells. (**A**) Polymorphic restriction enzymes used to distinguish the two parental alleles. SNP: single nucleotide polymorphism. The *IRAIN* lncRNA was reverse transcribed into cDNA using SSRT oligonucleotides. The PCR products were digested by polymorphic *Alu* I and *Sac* II. (**B** and **C**) Allelic expression of *IRAIN* lncRNA at the E site in KG-1 and KG-1a leukemia cells. gDNA: heterozygous genomic DNA. Note the single ‘A’ allele or the single ‘G’ allele expression of *IRAIN* lncRNA. (D) Parental imprinting of *IRAIN* lncRNA by tracking allelic expression in three families. Genomic DNA and cDNA were amplified from peripheral blood cells and the PCR products were sequenced for the A/G alleles. Note the monoallelic expression of *IRAIN* lncRNA from the paternal allele.

The expression pattern of the *IGF1R*/*IRAIN* locus is quite similar to the *Igf2r*/*Airn* imprinting locus in the mouse, where *Airn* is imprinted and the lncRNA *Airn* regulates in *cis* the allelic expression of the *Igf2r* coding RNA (43–46). We therefore examined if *IRAIN* uses a similar epigenetic mechanism to regulate genes. We first examined if *IRAIN* was monoallelically expressed in tissues. After screening several single nucleotide polymorphisms (SNPs) within the *IRAIN* locus, we took advantage of polymorphic *Alu* I and *Sac* II sites to examine allelic expression of the *IRAIN* lncRNA (Figure 2A).

Leukemia KG-1 cells are informative for both *Alu* I and *Sac* II. After restriction enzyme digestion, both the A and G (or C) alleles were detected in the genomic DNA (Figure 2B, lanes 2 and 5). In cDNA samples, however, only the ‘A’ allele was detected (lanes 3 and 6), indicating that *IRAIN* lncRNA is monoallelically transcribed in KG-1 leukemia cells. Similarly, KG-1a cells also expressed the *IRAIN* lncRNA in a monoallelic manner, as only the ‘A’ allele was detected in both the *Alu* I and *Sac* II sites (Figure 2C, lanes 3 and 6).

To determine which parental allele transcribes this noncoding RNA, we collected peripheral white blood cells from three families and tracked the expression pattern of the *IRAIN* lncRNA. In family A, the father carried both the A and G alleles and the mother had the G allele. The child was informative at the polymorphic site, carrying both the A and G alleles in genomic DNA. In the cDNA sample, however, we detected the expression of *IRAIN* lncRNA only from the A allele that was inherited from the father (Figure 2D, left panel), demonstrating that this lncRNA is paternally expressed and maternally suppressed. We also confirmed the paternal expression in two other families. In both family B and family C, the mothers were homozygous for the G allele, while the children were heterozygotes, carrying both the A and G alleles; only the paternal A allele was expressed (middle and right panels). Together, these data suggest that *IRAIN* is an imprinted gene, with the paternal allele expressed and the maternal allele suppressed.

### Allelic regulation of *IRAIN* by epigenetic mechanisms

**Figure 3.**
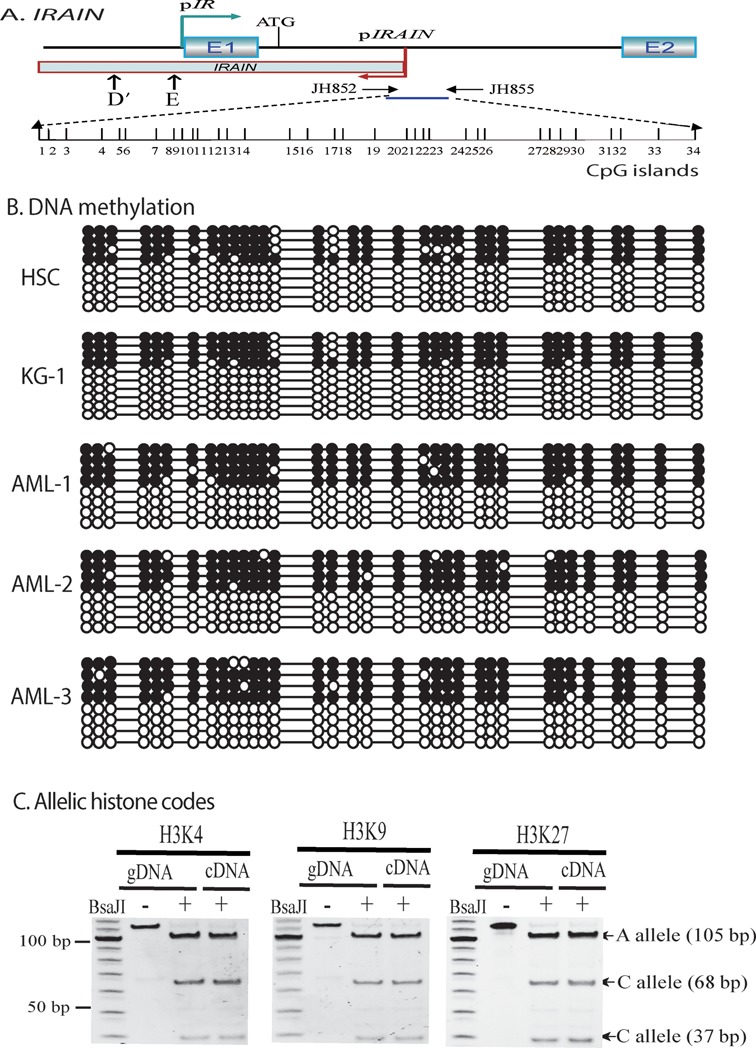
Epigenetic regulation of the *IRF1R*/*IRAIN* locus. (**A**) Schematic diagram of CpG islands in the *IRAIN* promoter. Vertical lines: location of CpG islands. (**B**) The status of CpG island DNA methylation in the *IRAIN* promoter. Genomic DNAs were extracted from leukemia cells and AML cells collected from three patients. After treatment with sodium bisulfite, the *IRAIN* promoter DNA was amplified and sequenced. Open circles: unmethylated CpGs; solid circles: methylated CpGs. (**C**) Allelic histone codes in the *IRAIN* promoter. Chromatin complex DNAs were pulled down by immunoprecipitation. After PCR, the two parental alleles were separated by polymorphic restriction enzyme *Bsa*J1 for histone methylation at H3K4, H3K9 and H3k27. Input: chromatin DNAs collected before antibody immunoprecipitation.

Imprinted genes are often regulated by differentially methylated regions (DMRs) in an imprinting control region (ICR) (26,47,48). As the *IRAIN* promoter is very rich in CpG dinucleotides (Figure 3A), we analyzed the status of DNA methylation in the *IRAIN* promoter. Genomic DNA was treated with sodium bisulfite to convert unmethylated cytosines into uracils while methylated cytosines are left unchanged. The treated DNA was amplified with methylation-specific primers, cloned in pJet vector and sequenced.

Using bisulfite sequencing, we found that the *IRAIN* promoter is hemi-methylated in normal hematopoietic stem cells (HSCs) (Figure 3B). A similar hemi-methylation pattern was also observed in the *IRAIN* promoter in KG-1 leukemia cells and in three AML samples. Although we could not find a SNP in this region to distinguish the two parental alleles in this DMR, the hemi-methylation pattern is typical of a DMR in an ICR, suggesting that the monoallelic expression of *IRAIN* may be associated with the status of DNA methylation in the gene promoter. However, K562 leukemia cells showed complete methylation in the promoter (Supplementary Figure S3). Thus, the epigenotype in the *IRAIN* promoter may be aberrantly altered in this tumor line.

We then utilized ChIP to examine the histone methylation in the *IRAIN* promoter. After immunoprecipitation using antibodies against methylated H3-K4, H3-K9 and H3-K27, PCR was used to amplify the chromatin DNAs. A BsaJ1 polymorphic restriction site, located ∼300 bp downstream of the differentially methylated promoter region, was used. We did not detect an allelic difference in the histone methylation products between the two alleles (Figure 3C), excluding histone methylation in the promoter as the determinant in the control of *IRAIN* allelic expression.

### Interaction of *IRAIN* lncRNA with chromatin DNAs

**Figure 4.**
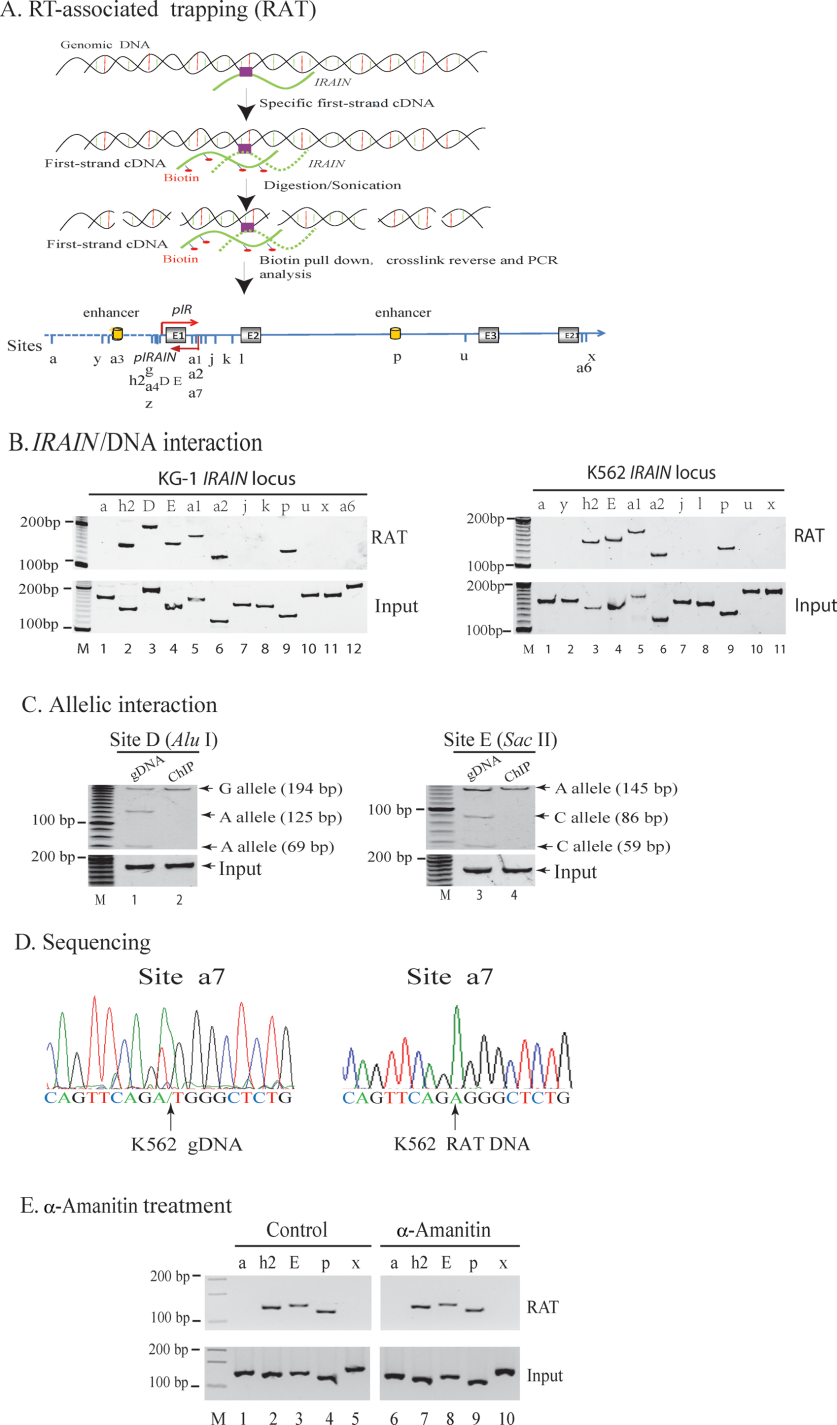
Chromatin DNA interaction of the *IRAIN* lncRNA. (**A**) Detection of *IRAIN* lncRNA–DNA interaction. A reverse transcription-associated trap (RAT) assay was used to detect the *IRAIN* lncRNA-specific interaction with *IGF1R* promoter DNAs. Alphabetic numbers: location of lncRNA binding sites. (**B**) *IRAIN* lncRNA–DNA interaction in KG-1 and K562 leukemia cells. Note the enriched binding of *IRAIN* lncRNA at *IGF1R* promoter (a4 and a5 sites) and intragenic enhancer (p site). Input: RNA–chromatin complex aliquots collected before RAT. (**C**) Allelic interaction of the *IRAIN* lncRNA with chromatin DNAs. Two polymorphic restriction enzymes *Alu* I and *Sac* II were used to distinguish the paternal and maternal alleles. Note the monoallelic binding of the *IRAIN* lncRNA. (**D**) Sequencing confirmation of allelic *IRAIN* lncRNA binding with the IGF1R promoter chromatin DNA. Left: K562 genomic DNA with both ‘A/T’ alleles; right: the *IRAIN* ChIP DNA with the single ‘A’ allele. (**E**) The lncRNA/DNA interaction after depletion of nascent RNA with α-amanitin. Cells were pretreated with the RNA polymerase inhibitor α-amanitin. After depletion of the nascent RNA, cells were fixed and used to detect DNA interaction with the RAT assay. The α-amanitin treatment does not interfere with lncRNA/DNA interaction.

As several lncRNA molecules regulate gene function by directly binding to their target chromatin DNAs (28–30,49,50), we determined if *IRAIN* lncRNA bound to *IGF1R* chromatin DNA, particularly the promoter region. We developed a ‘RAT’ assay that overcomes the high noise to signal background of existing methodologies to detect RNA/DNA interactions (Figure 4A). Cells were treated with formaldehyde to fix the structure of the lncRNA-chromatin conformations. The chromatin DNA-interacting lncRNA was then reverse transcribed into cDNA using strand-specific oligonucleotides and a thermo-stable reverse transcriptase in the presence of biotin-dCTP. After digestion with restriction enzyme or sonication to shear chromatin DNAs, the biotinylated *IRAIN* cDNA–DNA complex was then separated from other DNA–DNA products by streptavidin pull-down using paramagnetic Dynabeads (Dynal, Invitrogen) and analyzed by PCR using interacting DNA-specific primers.

Using this approach, we detected an interaction of *IRAIN* lncRNA with chromatin DNAs in the *IGF1R* promoter (Figure 4B, sites h2–a2, lanes 2–6) and in an intronic enhancer (site P, lane 9) in KG-1 cells. Taking advantage of polymorphic *Alu* I and *Sac* II sites, we observed that the *IRAIN* lncRNA interacted with chromatin DNA in a monoallelic manner, with only the A allele detected by both polymorphic restriction enzymes (Figure 4C, lanes 2 and 4).

A similar interacting pattern was also detected in the leukemia K562 cell line (Figure 4B, lanes 3–6, 9). By sequencing, it was also determined that these PCR products were derived from the A allele alone (Figure 4D, bottom panel) as compared with the genomic DNA that contains both the A and T alleles (top panel).

To further validate this chromatin DNA binding, we treated cells with the RNA polymerase inhibitor α-amanitin (40,41). As the half-life of pre-RNA is very short, usually on the order of minutes (51), we treated cells with α-amanitin (75 μg/ml) for 5 h. To examine the efficiency of α-amanitin treatment, we extracted chromatin RNA by sucrose gradient centrifugation (52) and examined target preRNAs and RNAs using RT-PCR. As previously reported (53,54), α-amanitin treatment not only permitted preRNAs to mature, but also significantly reduced the level of target RNAs (Supplementary Figure S4). After depletion of the pre-mRNA, the RAT assay revealed that it was the *IRAIN* lncRNA that interacted with genomic DNAs (Figure 4E). Further studies are still needed to exclude the presence of the trace amount of the *IRAIN* pre-RNA formed during transcription in the chromatin complex.

### Long-range intrachromosomal loop orchestrated by *IRAIN* lncRNA

Since *IRAIN* lncRNA interacts with both the *IGF1R* promoter and the intronic enhancer DNA, which are about 150 kb apart, we hypothesized that the *IRAIN* lncRNA may participate in scaffolding these long-distance DNA regions to form an intrachromosomal loop. We therefore used 3C (37) to assess the potential chromatin interactions between these two DNA regions.

**Figure 5.**
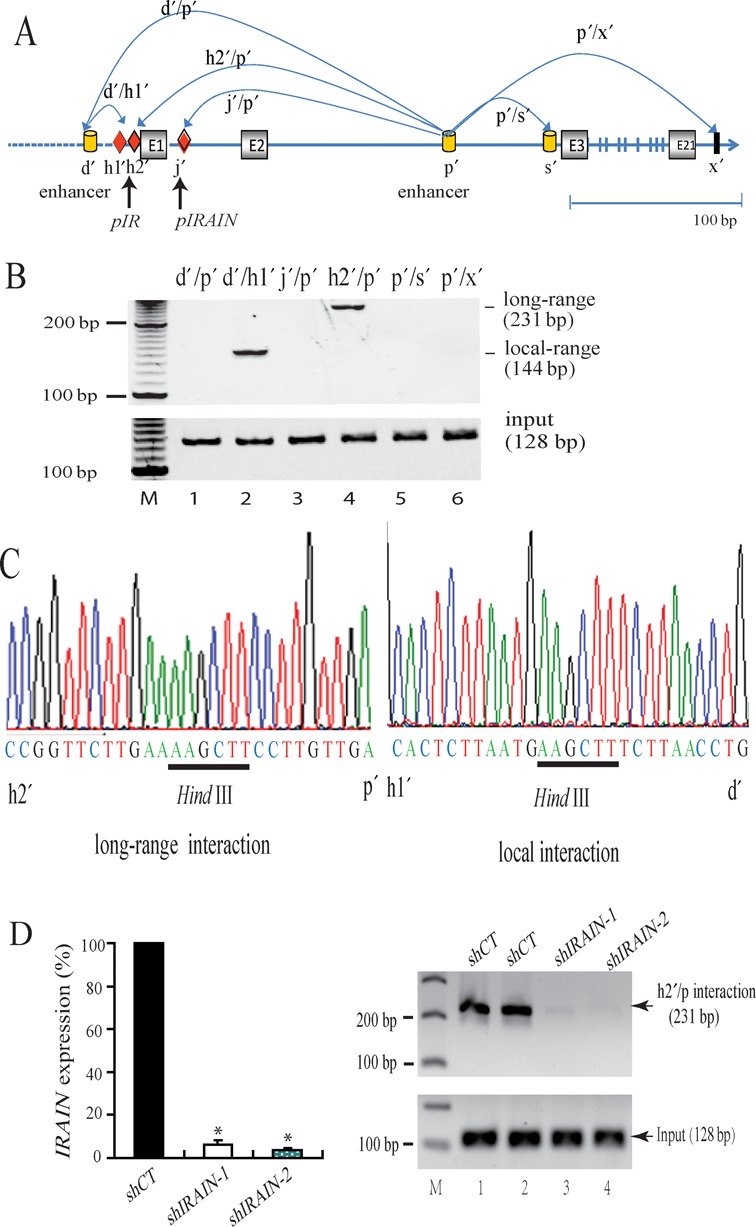
Intrachromosomal loop between *IGF1R* promoter and the intragenic enhancer. (**A**) Schematic diagram of the *Hind* III sites in the *IGF1R/IRAIN* gene locus used for the 3C assay. (**B**) Detection of chromatin interactions in KG-1 leukemia cells as detected by 3C assay. M: 100 bp markers, input: chromatin complex aliquots collected before 3C assay. (**C**) Sequencing confirmation of the 3C PCR products. Left: the *Hind* III site in the 3C DNA is flanked by DNA sequences from the *IGF1R* promoter and enhancer; right: local chromatin interaction. (**D**) *IRAIN* lncRNA knockdown abolishes the intrachromosomal DNA interaction. Cells were transfected with two shRNAs. After *IRAIN* knockdown, cells were fixed and used to detect DNA interaction with the 3C assay. Note the requirement for *IRAIN* in the maintenance of the *IGF1R* promoter/enhancer DNA interaction.

KG-1 cells were fixed with 2% formaldehyde, digested with restriction enzyme *Hind* III and then ligated with T4 DNA ligase to examine the remote interaction between these two DNA regions that are 150 kb apart (Figure 5A). Using primers from these remote regions, we found that the *IGF1R* promoter DNA interacted with the putative intronic enhancer DNA (Figure 5B, lane 4). In addition, a local DNA interaction was also detected between sites d′ and h′ (lane 2). Both interactions were confirmed by DNA sequencing (Figure 5C). ChIP assay also showed that both the *IGF1R* promoter (h2′) and the enhancer (p′) region sites were associated with the active enhancer marker, H3K4 methylation (Supplementary Figure S5). These data suggest that *IRAIN* lncRNA may be actively involved in the interaction of two remote regions of the IGF1R gene.

To further study the role of the lncRNA in this intrachromosomal interaction, we knocked down *IRAIN* lncRNA with two shRNAs. Both shRNAs significantly decreased the *IRAIN* lncRNA (Figure 5D, left panel). In the mock shRNA (shCT) treated cells, there was an interaction between the *IGF1R* promoter and the enhancer (Figure 5D, right panel, lanes 1 and 2). However, this long-range intrachromosomal interaction was abolished in the *IRAIN* knockdown cells (lanes 3 and 4). These data suggest the involvement of the lncRNA in the formation and/or maintenance of the long-range intrachromosomal loop.

### Downregulation of *IRAIN* lncRNA in hematopoietic malignancies

**Figure 6.**
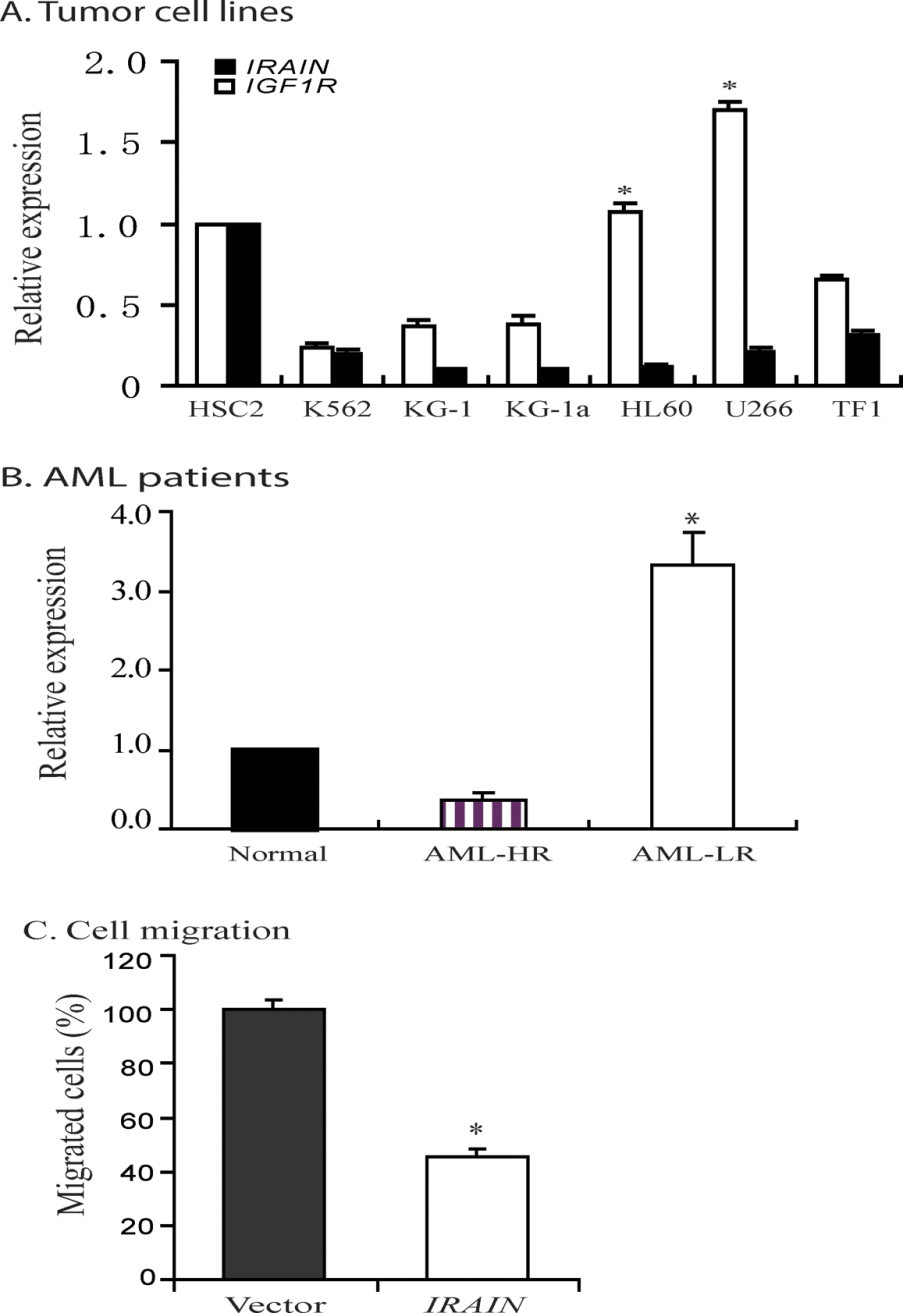
Downregulation of the *IRAIN* lncRNA in tumor cell lines and AML tissues. (**A**) Expression patterns of *IRAIN* and *IGF1R* in tumor cell lines. β-Actin was used in the internal PCR control for qPCR. Data are represented as mean ± SD. The relative expression was determined by normalizing the qPCR signals over that of normal HSC2 cells. *P < 0.05 as compared with the *IGF1R* coding RNA. (**B**) Expression of *IRAIN* lncRNA in AML patients. HR, LR: AML with high and low risk (AML-HR, *n* = 16; AML-LR, *n* = 18; and normal, *n* = 5). Data are represented as mean ± SD. *P < 0.05 as compared with the AML high-risk patients. (**C**) Inhibition of cell migration by 5.4 kb *IRAIN* lncRNA. Lentiviruses containing the full-length lncRNA and the vector control were transduced into MDA 231 cells. Stable cell clones were used to test the cell migration using transwell assay. Data are represented as mean ± SD. *P < 0.01 as compared with the vector control.

*IGF1R* is frequently overexpressed in human tumors. We were curious if *IRAIN* was dysregulated in tumors as well. We first used real-time PCR to compare the abundance of *IRAIN* lncRNA and *IGF1R* coding RNA transcripts in hematopoietic cell lines. Using a normal hematopoietic stem cell line HSC2 as a standard, we found that *IRAIN* was downregulated in leukemia cell lines as compared with the *IGF1R* sense coding RNA (Figure 6A).

We then collected bone marrow cells from high- and low-risk AML patients. Real-time PCR was used to quantitate the transcript abundance of both coding and noncoding RNAs. We found that *IRAIN* abundance was low in the high-risk group and high in the lower risk group (Figure 6B).

As a first step to explore the role of *IRAIN*, we virally expressed the full-length 5.4 kb *IRAIN* lncRNA in MDA231 tumor cells. The stable cell clones were then tested for their migration by transwell assay (35). As compared with the vector control, expression of the full-length 5.4 kb *IRAIN* lncRNA significantly inhibited tumor cell migration (Figure 6C). We also found that exposure of cultured cells to the chemotherapeutic agent cytarabine (AraC) enhanced *IRAIN* expression (Supplementary Figure S6).

## DISCUSSION

*IGF1R* is a validated tumor target for developing small molecule inhibitors and antibody therapies that block its tyrosine kinase activity. In examining the mechanism accounting for *IGF1R* dysregulation in tumors, we have identified a novel antisense noncoding RNA, *IRAIN*, that is expressed in antisense orientation and in a parent-of-origin-specific manner. The full-length *IRAIN* transcript is a 5.4 kb noncoding RNA; no large open reading frames can be identified using software prediction programs. Allelic expression of *IRAIN* is correlated with the status of CpG DNA methylation in the gene promoter (Figure 3B). We have not been able to find a SNP in the sequencing region to distinguish the two parental alleles. There is a BsaJ1 polymorphic site in the 3′-region of the promoter. However, the DNA sequence containing this BsaJ1 site is very GC-rich. After sodium bisulfite treatment, the fragment could not be amplified. Future studies using interspecific Mus spretus/Mus musculus F1 mice (45,55) may be used to examine the role of DNA methylation in *IRAIN* parental imprinting.

Gene ‘expression competition’ and ‘transcriptional interference’ have been proposed as mechanisms by which noncoding RNAs regulate allelic transcription. The best-studied examples are X chromosome inactivation and *Igf2r/Airn* allelic expression (47,56–59). Initiation of both imprinted and random X chromosome inactivation is dependent on a unique, noncoding RNA (*Xist*). The *Xist* lncRNA triggers the silencing of the X chromosome in *cis* through distinct domains (60) using a ‘chromosome coating’ mechanism (61). Similarly, short synthetic hairpin RNAs that are complementary to gene promoters are also able to modulate gene expression (62–64). In this study, we demonstrate that the *IRAIN* lncRNA is transcribed in an antisense orientation using a promoter located in the *IGF1R* intron 1. It is possible that *IRAIN* may use a similar ‘coating’ approach to regulate the transcription of the coding *IGF1R* RNA. By overlapping with the *IGF1R* promoter in antisense, the *IRAIN* lncRNA may directly compete with *IGF1R* in *cis* for the transcriptional machinery in the host. Downregulation of this lncRNA, as observed in high-risk hematopoietic malignancies (Figure 6), may relax the ‘transcription competition’ control and thus activate the *IGF1R* gene, leading to growth advantage and tumor progression. However, we found that both *IRAIN* shRNA knockdown and *IRAIN* overexpression approaches did not significantly affect the level of *IGF1R* RNA in treated cells (Supplementary Figure S7). It should be emphasized that both methods affect *IRAIN* lncRNA at the post-transcriptional level. It could be possible that like *Airn*, *IRAIN* lncRNA may use a ‘*cis*’ mechanism to regulate gene expression. If so, the overexpressed ‘*trans*’ lncRNA may not significantly affect the allelic expression of *IGF1R*. For this reason, a gene knock-out model may be needed to address a potential ‘*cis*’ role of *IRAIN* lncRNA.

Several lncRNAs regulate their target genes by directly binding their promoters and enhancers, including *Kcnq1ot1*, *Xist* and *HOTAIR* (28,29,65,66). *HOTAIR* can serve as a scaffolding factor, providing a binding surface to assemble histone modifying enzymes (67). In this study, we demonstrate that *IRAIN* interacts directly with regulatory chromatin DNAs of the *IGF1R* enhancer and promoter (Figure 5). While *HOTAIR* and *IRAIN* do not contain any similar sequences, they may share structural features that could allow them to regulate gene expression using a common epigenetic mechanism (68).

Long-range intrachromosomal interaction is a common epigenetic mechanism that juxtaposes a promoter with a remote regulatory element (34,39,69,70). For example, *Kcnq1ot1* lncRNA is a bidirectional silencer that regulates genes in *cis* over ∼1 Mb in the *Kcnq1* imprinting domain (29,30). KvDMR1, the region that carries the imprinting signal to control imprinting, is ∼200 kb away from *Kcnq1*. Our recent data demonstrate that using intrachromosomal looping, *Kcnq1ot1* recruits the histone H3K27 methylase EZH2, a component of the polycomb repressive complex 2 (PRC2), to the *Kcnq1* promoter target sequence, where the allele-specific histone H3-K27 methylation turns off the expression of the paternal *Kcnq1*. In *Kcnq1ot1* deficient cells, the absence of intrachromosomal looping leads to biallelic expression of *Kcnq1* (22). The data in this study demonstrate that *IRAIN* lncRNA is involved in the formation of an intrachromosomal interaction between the *IGF1R* promoter and a distant intragenic enhancer (Figure 6). Knockdown of this lncRNA abolishes this intrachromosomal interaction. Further studies are needed to delineate if this intrachromosomal interaction is also involved in the regulation of *IGF1R* expression in *cis* as in the case of *Airn* lncRNA in the IGF2R locus.

It is interesting to note that *IRAIN* is transcribed monoallelically. By tracking the allelic expression in three families, we demonstrate that *IRAIN* lncRNA is transcribed from the paternal chromosome, while the maternal allele is suppressed (Figure 2D). A single parental *IRAIN* allele is transcribed in KG-1 and KG-1a tumor cells (Figure 2B and C). This study thus adds *IRAIN* lncRNA to the list of monoallelically expressed transcripts (48).

Equally interesting is the finding that the expression of both the antisense *IRAIN* lncRNA and the sense *IGF1R* coding RNA is uncoupled in tumors. We found that *IRAIN* is monoallelically expressed (Figure 2). The *IGF1R* coding mRNA, however, is biallelically expressed (Supplementary Figure S8) in accordance with previously published reports (71,72). In several well-studied imprinting loci, the allelic expression of the sense and antisense RNAs is coupled via a *cis* transcription competition mechanism. For example, allelic expression of genes in the mouse *Igf2r/Airn* loci is coupled (56,73,74) and tightly associated with the status of DNA methylation in the *Airn* promoter (32) (Supplementary Figure S9, right panel). The maternal *Airn* promoter is hypermethylated and is silenced. Lack of the *Airn* lncRNA *cis*-competition leads to the active *Igf2r* transcription from the maternal allele. In contrast, the unmethylated paternal *Airn* promoter is expressed. The transcribed noncoding RNA silences in *cis* the *Igf2r* promoter (32,75). In the *IGF1R/IRAIN* locus, however, *IRAIN* is paternally expressed, but its sense *IGF1R* coding RNA is biallelically expressed (Supplementary Figure S9, left panel). Currently, it is unclear how these two RNAs are expressed in a divergent manner. Nonetheless, the fact that both *IRAIN* antisense lncRNA and *IGF1R* sense RNA are transcribed from the paternal chromosome without transcription competition or inhibition may provide a unique model to study imprinting mechanisms as seen in the *Igf2r/Airn* locus (56,74).

*IRAIN* is a newly identified lncRNA. We know very little about the specific function of *IRAIN*. Expression analyses reveal that *IRAIN* is downregulated in leukemia cell lines. The AML low-risk patients had greater *IRAIN* lncRNA expression than did the AML high-risk patients. Viral expression of the 5.4 kb lncNRA inhibits tumor cell migration, suggesting a tumor suppressor function. However, the low-risk patients also had greater *IRAIN* expression than did the normal subjects (Figure 6). The low-risk patients still exhibit the malignant leukemia characteristics and are prone to convert to high-risk AML. Thus, further studies are needed to address the specific role of the *IRAIN* lncRNA in tumors.

In summary, we have identified *IRAIN* as a novel lncRNA that is downregulated in leukemia cell lines and in patients with high-risk AML. The lncRNA is imprinted and directly interacts with the *IGF1R* promoter and enhancer chromatin DNA sequences. *IRAIN* also participates in the orchestration of an intrachromosomal loop between the IGF1R promoter and enhancer. Further studies are needed to delineate the specific role of this newly identified lncRNA in the upregulation of the IGF pathway in malignancies.

## SUPPLEMENTARY DATA

Supplementary Data are available at NAR Online.

SUPPLEMENTARY DATA

SUPPLEMENTARY DATA
