# Supplementary material for: A novel antisense long noncoding RNA within the IGF1R gene locus is imprinted in hematopoietic malignancies
Source: Nucleic Acids Res. 2014 Aug 4;42(15):9588–601. doi: 10.1093/nar/gku549 (PMC4150754; doi:10.1093/nar/gku549)
Supplement: SUPPLEMENTARY DATA [file supp_42_15_9588__index.html]

A novel antisense long noncoding RNA within the IGF1R gene locus is imprinted in hematopoietic malignancies — A novel antisense long noncoding RNA within the IGF1R gene locus is imprinted in hematopoietic malignancies — SUPPLEMENTARY DATA 

# A novel antisense long noncoding RNA within the *IGF1R* gene locus is imprinted in hematopoietic malignancies

## SUPPLEMENTARY DATA

**Files in this Data Supplement:**

- SUPPLEMENTARY DATA
- SUPPLEMENTARY DATA
