# Supplementary material for: A novel antisense long noncoding RNA within the IGF1R gene locus is imprinted in hematopoietic malignancies
Source: Nucleic Acids Res. 2014 Aug 4;42(15):9588–601. doi: 10.1093/nar/gku549 (PMC4150754; doi:10.1093/nar/gku549)
Supplement: SUPPLEMENTARY DATA [file supp_gku549_nar-01050-y-2014-File004.pdf]

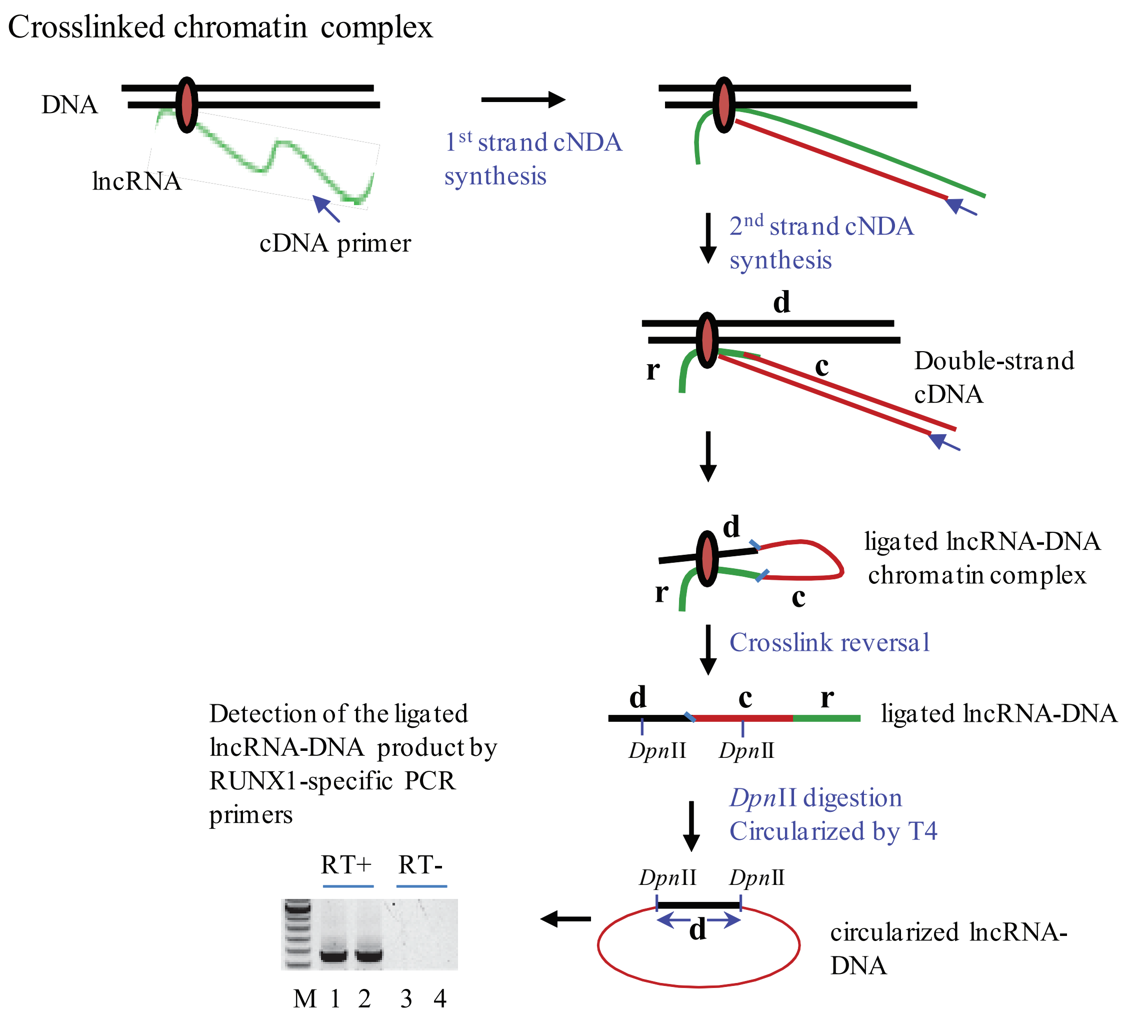

Figure S1. A modified R3C method to detect the chromatin DNA-interacted RNAs

IRAIN lncRNA sequence (5366 bp):

(TAGCAAGATATCAGCTCGTATGCCGTCTTCT**GGGG**)
cagcctttctgaattgccccggtgatggggccatacaacctccgctgcctcctccttccccgcctcactcgtgaaggctcagtcgtgatttttcaa
agttaatcggcaccacctgcataccctcggcaaccattactcaccaacacgccctcaccgttcccagtcccagttctgcgacatttaaacttttaat
cgtgttcaaattaccctaccatcttgaatgtaccagtaagccacactttcaactttttcagaagaataaaccccccacctcctccggccgtagacac
cggggggagttctcgctccccgcatccccgcgcttccctactaaccccacccgacaccaccaaaacaaaagctatcagattgcaaaagacagcgaa
tgtggagcgacactgcttattaactacgtacaaaggaagtgcggggcggcagaagagagggcgacacgacgccgaccacaacaaaaccaaggccagg
gctccgcgctcgcaaaacacaacaccctccccgcgccgccgcagaccctggcgctgaaaccacagcgtacgacacctctcgccagggacgggcgtct
aggtgtccccgtgaggcggcgactacaccccgcgctgcgagcacgtgtgtcggaatgcacgtcggctccgaccacacacccaccaacgcacactcgg
atgcggccctagtacccctccatcccccggcccgggagaaactgggagaccatcccagagccctctgaactggggacccagtggcgctccccggaag
agacaagctgaggaagactcccggcctcgcacttcccggggggctgcccagagagcgcttcccctcaccccaccgcccagccccgggacgccggggg
caggtaacccaacgaggagcgggccaaagtgacgggcaaggccaggaacccgaggagcccgaggggcaaaagcacgttgggcgacgggaggcagcga
cgggcgcttggagacgtgtcctggggcttcggggcctctccgcagggcacctccccccctcgagggcaactcgcaccccgcgggccgggcggctgccc
gctccccactgcccggtcagccccagcagcggagacgcggtgcggcccagggtgctgtcgctgctgcctgcgcgccgcaggggatcagcgctgtgtg
cggagaggctcttgcgcatggaaggggcgaggtgcgggtcgcggggcccgggaaccgcggagcccgccccggcaccaacttccctaccgagtcctcg
cgcctagccgggaaacacggctgggctgtcaggacggagaaagagaaaagcccttcccgcgcggggatgcgcggagccagggcccgcggggctccag
acgaacgagcccgggtcccgggtccgctgcagcctccctcccggcagagccgccctgcgccccaccccgccgctcccgagcccgggggcccgacagc
tgcgacggtggcaactcgggtttcgcaaacagggcgcagcccctcggaggaaaagttcccgcagtggccgcgggcggcgggcacatactcac**TTTCT**
CCACTCGTCGGCCAGAGCGAGAGCGCGGCGGAGAGAAACAGGAGCCCCCACAGCGAGGTCGGGGACCCTCCTCCGGAGCCAGACTTCATTCCTTTTA
TTTGGGATGAAATTCCCCTTTCTCAAAAAAAAAAAAAAAAAAAAAaaagaaaagaaaagaaaaaaaggaaatgaaaggaaacaagtctcaaactcag
tcttcgctcccctccaaaaacaagggcgaaggaaacaatactccgaaggcggcgccgccggggccctccgggtgcgcggacggggcgggcggcgcg
gggcggcccctcagcgccggcagcagcggcccagggctcggcggagtcgggaacccgaggcgcgccggggcgggctgtcggcgtcgtcggcctccat
tttcaaacccggagaggcaggaagggggaaaactgcaaagaaagggggcaaagcccaaatctgcctaggcgaggaaaaacaagcccggccctggcc
gcgcgggaggcggccggacgcccgagcttccgccgcgggagggcgcgacgcagttcgcaagatcgccccgaagtccgggtcacaggcgaggccggcg
aggggcagaaacgcggagtcaaaatgaatgagcggctccccctcctcctcctcctcctggctccgctcgccgccgcctcctccctcagcgccgccgc
cgccgcggcgagctccttcccaaatccaggacacacacaaagcggcgggccggccgcgccgcgctcagcactcccgccgcgccgcccggctctgcgc
cgccgcccgccccgcgccggcccccgcccggcccccgcccgccgccgcctgctctgcgcgggggctcc**ggctcgctgaaggtcacagc**cgaggcgag
gcgcgccgtggagctgcccccgcgcgggaggggcgtggagggcgcgggg**GGATCC**ggggagcggggccgagggtctgcgagcgccggccgtgggcag
gctcgctggcggcgtccctctcgctctcccgcctctctcgagttcgcctggtgcgctcgctcctcctcgggtttttcccttttttttccgctcag
cggagttaatgc**tggtaaacaagagccccagcct**cgccgcgccgccgccgccgctgccacacactgggggctcgcgcgcgcgcgcgcgccctggccc
gcgcacacgcgcccgctcgcacactcgagcggccaccgccgcagcagcggcgcgccgagggcccggcccaggccggcgcgcgcaaaaaaccggagcc
cggttccccaagacgtgcggagcggagcccgggcgtggggcgcgagactgcgaggcgccgcccccatctcccgccccaccgcctcccgcgcgcacac
agagacacgcgcgcgtgcccacccgctcccgcccccaccgcactgcgccggccaccccgcacgcgccctcccctcccgcccgactccaaaaacaatg
cccccgccgccggtccggtctcggcggggcaggggtgggtagccagggaaaggctaggcaggggaggggtgggcaggagaggggaggggtgggcggg
gagtgcggggctgggagaggttcattgaaaacaacaacagcgcggctggaaagcgcgtttccccggttcttgaaaagcttcattgtttcttgcagct
gttgcaaaataaataaatgcgtgcatgcggcatttttttcgctgcgtactggaaagcccgaacgtggcggaaggggcgcacctgggagaaaggggac
cccccccccaggctactacgccgggcactttcacgccaggctggggtgcccgcggcgttgcgaccgtggagcccggagtttgggggctcagggggag
cggagggggaagggcgcggcccgtgtggctcggcccagagtggctcgaggagagaggaccccgccgcccctcgcgggccccgcgccccggaaggggg
gcccagacaggcagcgccgcgtgccccgggcgcgtccccgcggcggccgccagagggcgcggcgggcgctggggagggcagcccgcggagagtcgcc
tccctccctccgggggaggaaatgtgggggcccgggctgggggccgactcggctgggtcgcgctggcgtggcctcccggaccagccgcccgccctga
gcgctctccgcggcctcgcgcagctcagggctccaccacgtgggcggctgcggggagcggccggggcgcgtgcacgctgcgggtctccgaagctcgg
ggggcgtgtccgggtcccggggcgccgaggagggggtggaggctggggccgggaagcctcccttggccgcggtcccgacccgggaacacgccgcatg
cactcgggatcagcggctactgcggaaacccacccgcacctgtctcgggtcctcccaggcacgttcctcttgcttcgtttgctacgtaagtgactgg
aaaccagtaccgaccctaagtctccgcggttgttttcttgaatgtgcaggaggagaggtttccccaaagcgagactcccctaggactgccatctaat
gaataaatgctgcggagaccaggtcctcaaagagggaaacagcccagcagtggcaggagagaggagtttgaaatacgtggacttttacaaacagtga
ttggaccatgtgtcgcccttaatgtggtccggtttcatgactcaggctaattgatgccagtaataaattttttaagatagctaataaaagagttgt
gaattgtcgagataagaagtggttgtctcaaacaatcgggtaggatggtagaagggacctgatgtaaaggaaggtagtaggtttcttttactctcta
attctaaaatacttctgcattagaacgtgtatccatcgttcgtaaataatttttaagagctgaaaacaagtcaaaagctatcataacggaaactta
gcccttttttctaaagttgtttcctagatcattttcctccttggaaatttctagctcaattcagagtcagttgtatagaaagaaaataaaccttgt
tttacctaagaaagttattcatatgatgggatggttttgtttaactgaaattcagcttgatttttaaataaagagtttcagttttggatgttcctag
atttaacagtgaaataaatttattagaatcttaaaatgtattatttaaaaattaggcactacacatgaagaattaaagtccagtctactaattttta
aatatttcttaagttctctgcatgaggttggtcttgattcctgcagatccactctggagttaaatgcaaaggaatattgatatataatttcattata
cttgatttcagaatttttaatacatacttgtaagacaaaaatttagagctgatttttaaaaggctagtgctaatataaaatcttcaaatactaccca
atgtattttagtattagtaataaatatgcctgtttatttgtccccaagaacataaagaggtctatagaattttaaatgtatataggttgttgtag
gggttgtaggatttgtttttgtgtgttttttttttactaaaatgatttttatttgtgaatttttttaaatgtttgtgttttcctgaagtagtaggaaa
tcaatcgtattaagttattttaaacagtgaagtgggagatgtatcattatatagcttaatatttagggaaaaaaaaggaacaaaaatttaggttgtc
tttacgctctaacttgaccacatggacaaaggaggtgatttgtatttcttcaatgattatattttacatacacaagtcaaacttatttttcaacct
tgaaataaaatcatgtagacaaaaaaaaaaa

Figure S2. *IRAIN* lncRNA sequence determined by 5’-racing

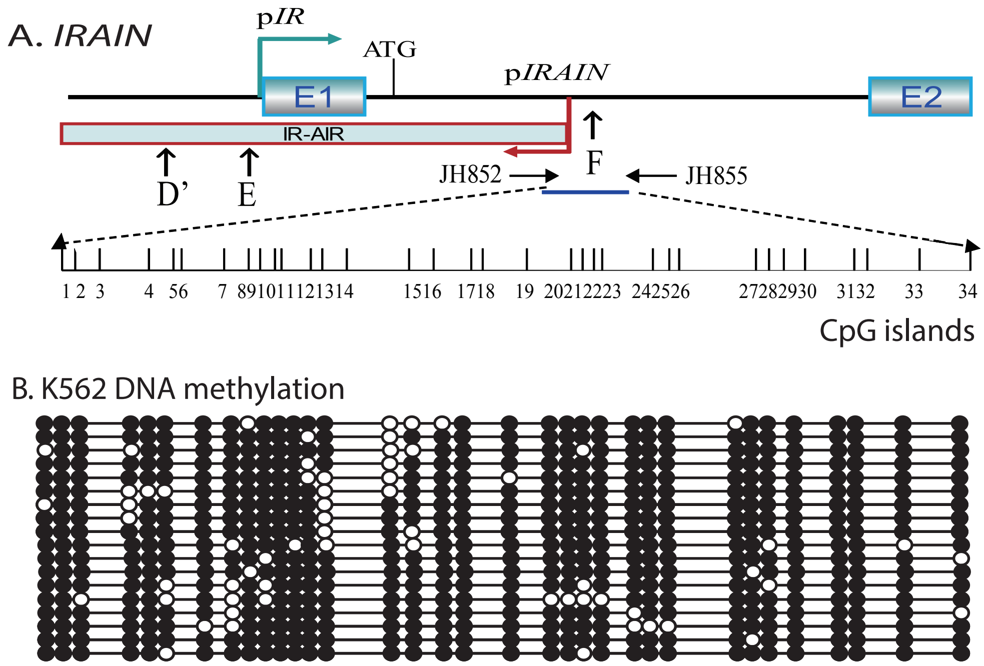

Figure S3. Fully methylated *IRAIN* promoter in K562 cells

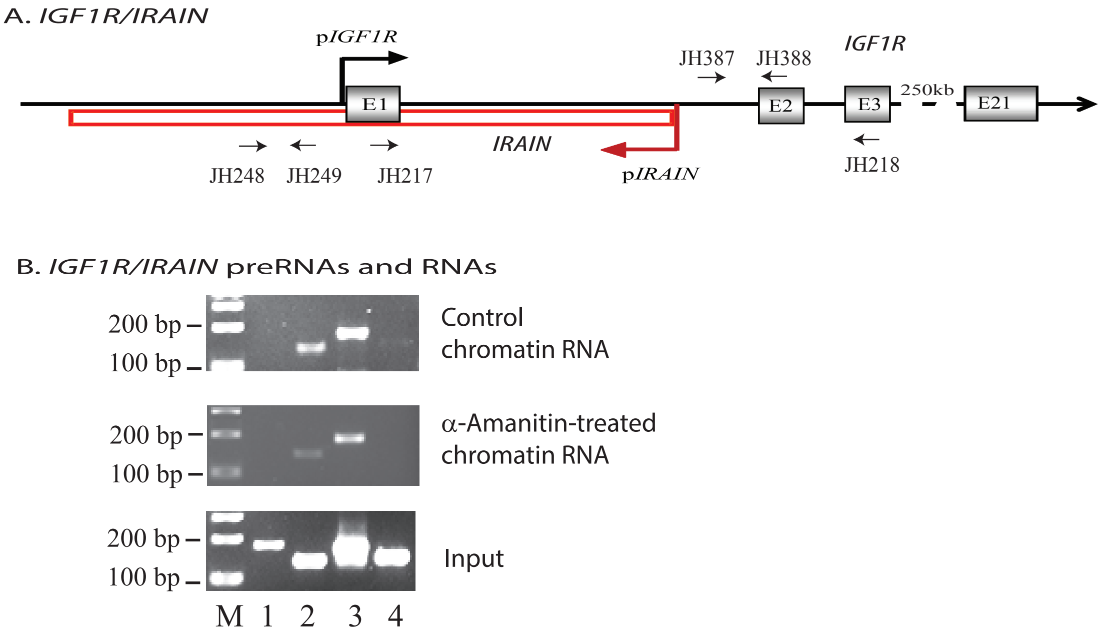

Figure S4. Chromatin-bound RNA in *a*-amanitin treated Kg-1 cells

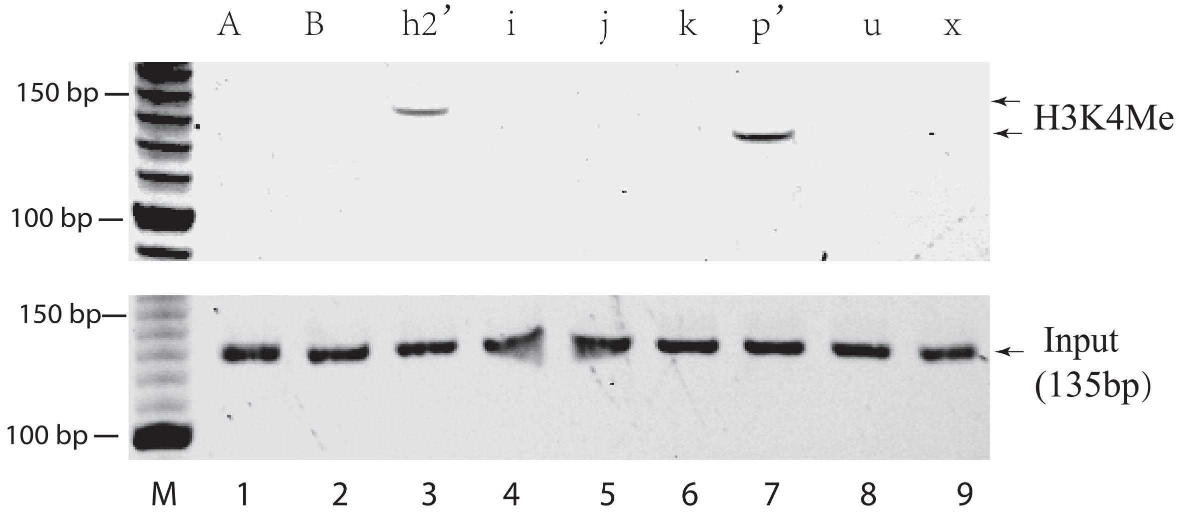

Fig.S5 Active chromatin marker H3K4Me in the ***IGF1R*** promoter and the enhancer

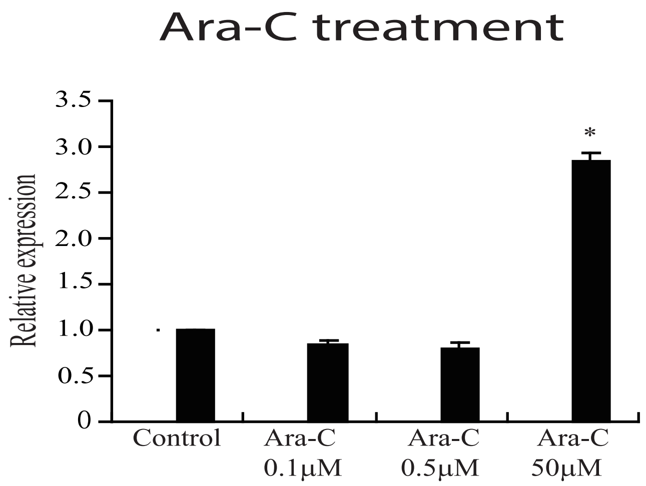

Figure S6. Upregulation of *IRAIN* lncRNA by Ara-C

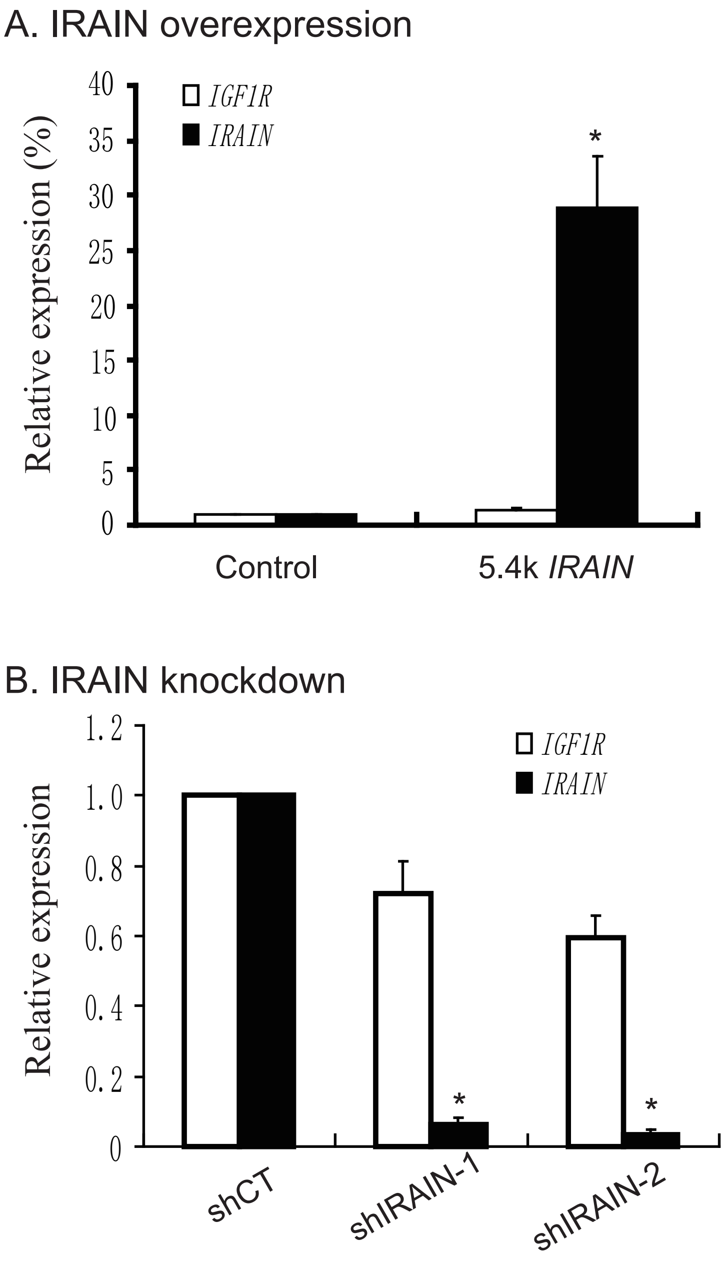

Figure S7. *IGF1R* expression in IRAIN overexpressed and knocked-down cells

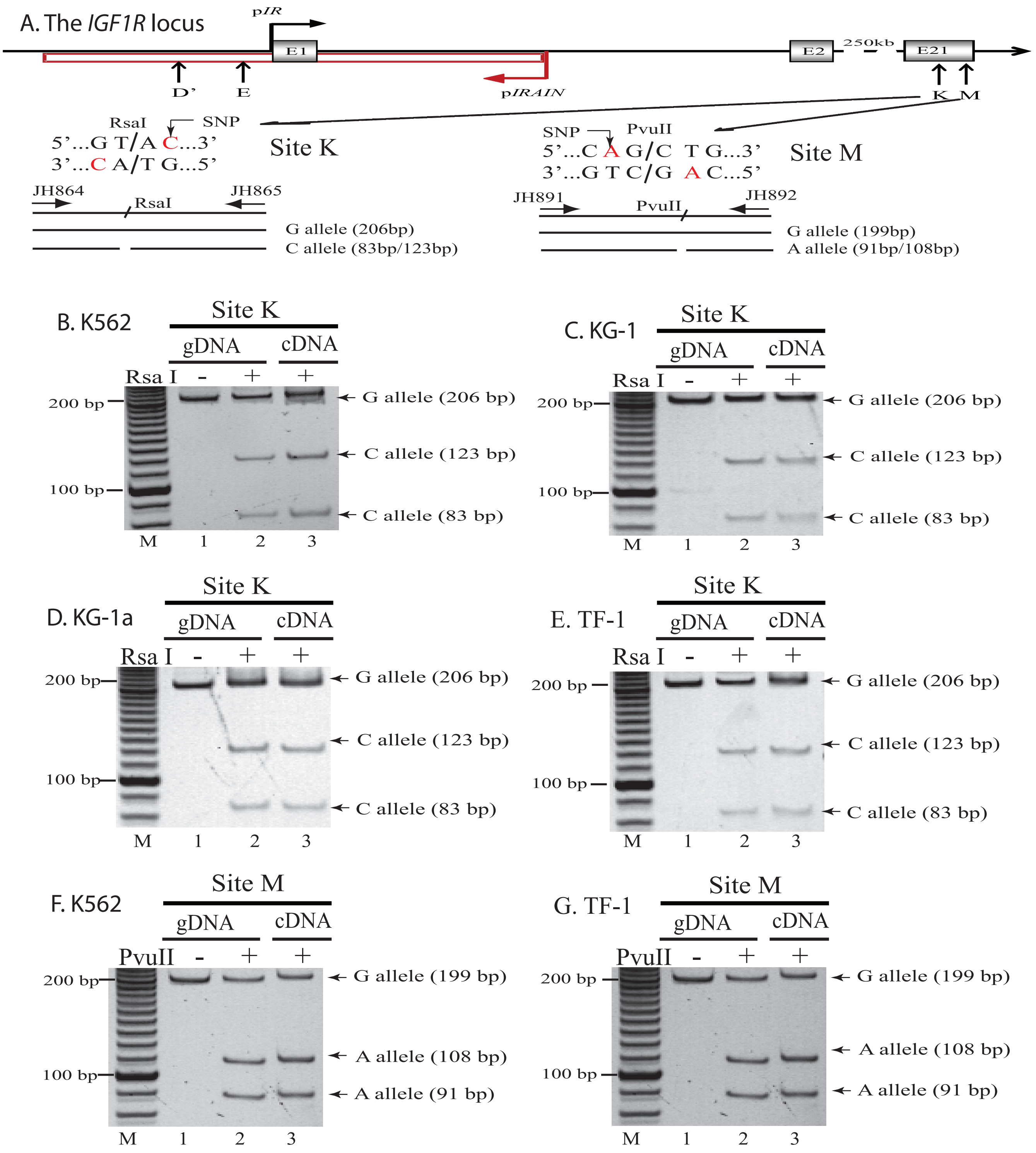

Figure S8. Biallelic expression of the *IGF1R* sense coding RNA

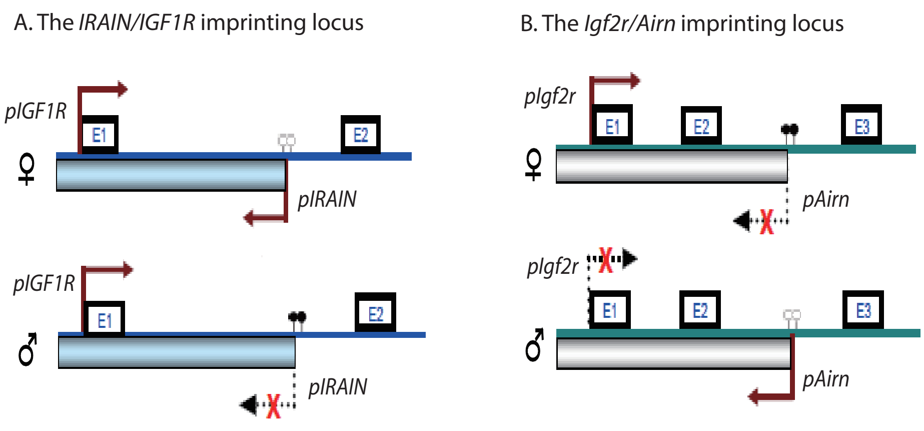

Figure S9. Allelic expression of the human *IRAIN/IGF1R* locus and the mosue *Igf1r/Airn* locus
