# Supplementary material for: A novel antisense long noncoding RNA within the IGF1R gene locus is imprinted in hematopoietic malignancies
Source: Nucleic Acids Res. 2014 Aug 4;42(15):9588–601. doi: 10.1093/nar/gku549 (PMC4150754; doi:10.1093/nar/gku549)
Supplement: SUPPLEMENTARY DATA [file supp_gku549_nar-01050-y-2014-File003_Updated.doc]

**SUPPLEMENTAL INFORMATION**

**A novel antisense long noncoding RNA within the *IGF1R* gene locus is imprinted in hematopoietic malignancies**

Jingnan Sun, Wei Li, Yunpeng Sun, Dehai Yu, Xue Wen, Hong Wang, Jiuwei Cui, Guanjun Wang, Andrew R. Hoffman, Ji-Fan Hu

**Figure legend**

**Figure S1. RNA-guided Chromatin Conformation Capture (R3C).** RT-: non-reverse transcriptase control; M: 100 bp marker; d: chromatin DNA; r: lncRNA; c: cDNA; horizontal arrows under the ligated DNA: primers used to detect the unknown R3C products.

Cells were cross-linked with 2% formaldehyde to fix the chromatin DNA/RNA complex structure. DNA-binding RNA was reverse-transcribed into double stranded cDNA with biotin labeling. After digestion with HindIII, the lncRNA-converted double strand cDNA was ligated to adjacent genomic DNAs by T4 DNA ligase. The biotinylated cDNA-DNA complex was separated from other DNA-DNA products by streptavidin/paramagnetic Dynabeads pull-down and was digested further by *Dpn*II. The *Dpn*II-digested complex was circularized by T4 ligase and amplified with *IGF1R* promoter-specific PCR primers. The R3C products were isolated and sequenced to identify the RNA molecules that interact with the *IGF1R* promoters (Zhang et al, Journal Cell Biology revision).

**Figure S2. *IRAIN* lncRNA sequence.** The *IRAIN* lncRNA was cloned from AML cDNAs by 5’- and 3’-RACE PCR, cloned into pJet vector (Thermo Fisher Scientific, MA), and joined as the full length lncRNA by a two sequential cloning into a lentiviral vector for sequencing. Underlined letters in red in parenthesis: 5’ RACE primer with four Gs that were annealed with the Cs at the end of the newly synthesized cDNAs; Capital blue letters: the complementary strand of the *IGF1R* exon 1. The *Bam* H1 (GGATCC) used to join the two RACE products as the full length *IRAIN* lncRNA was underlined in red capital letters. Two primers covering the *Bam*H1 site (underlined in green) were used for the 5’- and 3’-RACE. Two RACE products were then joined together by *Bam*H1 to construct the full length *IRAIN* lncRNA.

**Figure S3. Aberrant DNA methylation in the IRAIN promoter in K562 cells**.

1. Schematic diagram of CpG islands in the *IRAIN* promoter. Vertical lines: location of CpG islands.
2. B: Aberrant DNA methylation in the *IRAIN* promoter. K562 genomic DNAs were treated with sodium sulfite and the *IRAIN* promoter DNA was amplified and sequenced. Open circles: unmethylated CpGs; solid circles: methylated CpGs.

**Figure S4. Chromatin-interacting *IGF1R* and *IRAIN* preRNA and RNAs.**

1. Location of PCR primers used to amplify the *IRAIN*/*IGF1R* preRNA and mature RNAs. p*IRAIN*: *IRAIN* lncRNA promoter; p*IGF1R*: *IGF1R* coding RNA promoter.
2. KG-1 cells were treated with 75µg/ml α-Amanitin (Sigma, MO) for 5 hours to block nascent mRNA synthesis. After depletion of pre-mRNA, cells were collected and chromatin-interacting RNAs were extracted using the sucrose gradient centrifugation method. RT-PCR was then used to detect preRNAs and mature RNAs of *IGF1R* and *IRAIN*. M: 100bp marker; lane 1: *IGF1R* mature mRNA amplified by primers JH217/JH218; lane 2: *IRAIN* lncRNA by primers JH248/JH249; lane 3: small nuclear RNA(snRNA) U2 as the positive control amplified by primers JH234/JH235; and lane 4: *IGF1R* preRNA amplified by primers JH387/JH388. Input: PCR controls (lanes 1-3: total RNA collected before sucrose gradient centrifugation; lane 4:K562 genomic DNA). Note the weak PCR signal of *IGF1R* preRNA in the untreated control chromatin RNAs.

**Figure S5. Active chromatin marker H3K4Me in the *IGF1R* promoter and the enhancer.** Chromatin immunoprecipitation (ChIP) was used to measure the active chromatin maker, histone H3 lysine 4 methylation (H3K4Me). Cross-linked DNA-protein complexes were immunoprecipitated with antiserum against trimethyl-H3-K4 (#ab8580, Abcam, MA). Chromatin DNAs released were amplified by PCR with specific primers covering the promoter and enhancer of *IGF1R*. Input: genomic DNA collected before antibody precipitation. Note the H3K4Me enrichment in the promoter (h2’) and enhancer (p’).

**Figure S6. Ara-C upregulates *IRAIN* lncRNA in K562 leukemia cells.** Data are represented as mean+/-SD. * p<0.05 as compared with the controls. K562 cells (4×10^5^) were seeded in 6 wells in RP1640 Media. After overnight incubation, cells were treated with Ara-C (at concentrations of 0.1, 0.5, and 50 µM). After treatment for 48 hours, cells were collected and total RNA was extracted using RNeasy Kit (Qiagen, CA) for gene expression using qPCR.

**Figure S7. Expression of *IRAIN* and *IGF1R* in *IRAIN*-overexpressed and shRNA knocked-down cells.**

1. Gene expression in IRIAN-overexpressed cells. Lentivirus containing the full length 5.4 kb IRAIN lncRNA was used to transfect MB-MDA231 cells. Stable clones were collected for qPCR analysis. β-Actin was used the internal PCR control for qPCR. Data are represented as mean+/-SD. The relative expression was determined by normalizing the qPCR signals over that of control cells. * p<0.05 as compared with the control group.
2. Gene expression in IRAIN knockdown cells. IRAIN lncRNA was knocked down with two shRNAs in MB-MDA231 cells. After IRAIN knockdown, cells were collected for qPCR analysis. shCT: control shRNA; shIRAIN1-2: IRAIN shRNAs. Data are represented as mean+/-SD. * p<0.05 as compared with the shRNA control group.

**Figure S8.** **Biallelic expression of the *IGF1R* sense coding RNA**.

1. Polymorphic restriction enzymes used to separate the two parental alleles. SNP: single nucleotide polymorphism. p*IRAIN*: *IRAIN* lncRNA promoter; p*IR*: *IGF1R* coding RNA promoter. The *IGF1R* sense mRNA was reverse transcribed into cDNA for PCR. The PCR products were digested by polymorphic *Rsa* I and *Pvu* II.

B-G. Biallelic expression of *IGF1R* sense mRNA in K562, KG-1, KG-1a, and TF1 leukemia cells. gDNA: heterozygous genomic DNA. Note that both the “G/C” and “G/A” alleles were expressed in all leukemia cell lines, where the *IRAIN* antisense lncRNA was imprinted.

**Figure S9. Allelic expression of genes in the human *IRAIN/IGF1R* locus and the mouse *Igf2R/Airn* locus.**

1. Divergent allelic expression of the human *IRAIN* lncRNA and *IGF1R* coding mRNA. *pIRAIN*: *IRAIN* lncRNA promoter; *pIGF1R*: *IGF1R* promoter; black circle: methylated CpG islands; open circles: unmethylated CpG islands. Note the monoallelic expression of the *IRAIN* lncRNA from the paternal allele, while the *IGF1R* coding mRNA is biallelically expressed. Thus, the allelic expression of these two sense and antisense RNAs is uncoupled.
2. Coordinated regulation of allelic expression of the mouse *Igf2r* coding RNA and Airn noncoding RNA. *pIgf2r*: the mouse *Igf2r* coding RNA promoter; *pAirn*: the mouse *Airn* noncoding promoter. The allelic expression of these two sense and antisense RNAs is coupled and coordinated by DNA methylation in the *Airn* promoter located in the *Igf2r* intron 2 region. The maternal *Airn* promoter is methylated and silenced, leading to the expression of the maternal *Igf2R* coding mRNA. In contrast, the paternal *Airn* promoter is active due to the absence of DNA methylation. Expression of the *Airn* noncoding RNA silences the *Igf2r* coding RNA using a *cis* coating-competition mechanism.

**Table S1. AML patients in the High risk and Low risk groups**

|  | |  |  |
| --- | --- | --- | --- |
| Patient characteristics | High risk | Low risk | p-value |
| N  Age (yrs) | 16  44.7±12.9 | 18  40.4±12.0 | 0.319 |
| Gender | 9 | 10 | 0.968 |
| White blood cell (10^9^/L) | 17.0±9.8 | 14.4±7.9 | 0.406 |

**Table S2. Characteristics of 34 acute myeloid leukemia patients at diagnosis**

| Patients  Groups | No. | Gender | Age，yrs | WBC，  ×10^9^/L | Cytogenetic  data | Molecular genetic data |
| --- | --- | --- | --- | --- | --- | --- |
| Low-  Risk | 1  2  3  4  5  6  7  8  9  10  11  12  13  14  15  16  17  18 | M  F  M  M  F  M  F  M  M  M  F  M  F  F  M  F  M  F | 30  42  54  25  32  23  44  59  65  26  36  32  47  40  32  51  38  47 | 2.8  3.9  5.6  8.1  8.8  9  9.2  9.4  10.2  15.6  16.7  18.8  20.7  20.9  23.2  24.8  25.6  26.3 | *Normal*  *Normal*  *Normal*  *-*  *-*  *Normal*  *t(8;21)*  *Normal*  *Normal*  *t(8;21)*  *Normal*  *-*  *t(8;21)*  *inv(16)*  *t(8;21)*  *Normal*  *Normal*  *t(16;16)* | *CEBPA，no FLT3*  *NPM1，no FLT3*  *NPM1，no FLT3*  *RUNX1-RUNX1T1*  *RUNX1-RUNX1T1*  *NPM1，no FLT3*  *RUNX1-RUNX1T1*  *CEBPA，no FLT3*  *CEBPA，no FLT3*  *RUNX1-RUNX1T1*  *NPM1，no FLT3*  *CBFB-MYH11*  *RUNX1-RUNX1T1*  *-*  *RUNX1-RUNX1T1*  *CEBPA，no FLT3*  *NPM1，no FLT3*  *CBFB-MYH11* |
| High-  Risk | 1  2  3  4  5  6  7  8  9  10  11  12  13  14  15  16 | M  M  F  F  M  F  M  M  F  M  F  M  F  M  F  M | 28  53  26  56  35  68  29  55  47  58  41  62  34  38  42  44 | 3.5  4.2  6.8  9.0  9.7  10.5  11.8  13.0  16.7  18.2  23.4  25.2  27.1  29.6  30.8  32.1 | *-7*  *inv(3)*  *del(5q)*  *Complex*  *Complex*  *inv(3)*  *complex*  *-7*  *Complex*  *t(4;11)*  *t(6;9)*  *t(4;11)*  *complex* | *MLLrearranged（FISH）*  *MLLrearranged（FISH）*  *MLLrearranged（FISH）* |

-: Data for karyocype analysis were not available.

**Table S3. PCR Primers used in the study**

| Primer name Primer Number Primer sequence（5’-3’） | | | | | | | | | |
| --- | --- | --- | --- | --- | --- | --- | --- | --- | --- |
| Mapping | | | | | | | | | |
| A：forward  A：reverse  B：forward  B：reverse  C：forward  C：reverse  D：forward  D：reverse  E：forward  E：reverse  F：forward  F：reverse  G：forward  G：reverse  H：forward  H：reverse  I：forward  I：reverse  J：forward  J：reverse  C1：forward  C1：reverse  C2：forward  C2：reverse  C3：forward  C3：reverse  H1：forward  H1：reverse  H2：forward  H2：reverse  H3：forward  H3：reverse | | JH333  JH334  JH375  JH376  JH410  JH411  JH248  JH249  JH780  JH781  JH583  JH584  JH385  JH386  JH246  JH247  JH491  JH482  JH463  JH464  JH377  JH378  JH402  JH403  JH404  JH405  JH485  JH486  JH487  JH488  JH489  JH490 | | | | | | gcagaaaatagaagctgcaatcc  tgctgctgcaggtctattacaaa  cagagcccatcctaaaatcacc  Gggcagtgtttctctgaatgtg  Cgattgatttcctactacttcagga  gcctgtttatttgtccccaaga  cgacacatggtccaatcactgtt  agactcccctaggactgccatct  gtttccgcagtagccgctgat  Ctgcgggtctccgaagctc  acgcatttatttattttgcaacagc  agaggttcattgaaaacaacaacag  gccgacgagtggagaaagtg  Ctgcgacggtggcaactc  gggagtcttcctcagcttgtctc  acacccaccaacgcacactc  Ccttcttgaaggtttttgggatt  Gacaaatcactgtcaaccagca  Tgccgagggtacctggtcttag  Ttctgctctaacacctccagattc  ttaacctgaggcccaagtatgg  Cacttcaaacagcaatttttcacc  agagactaac caccgcaaat g  aaatggaaactggaggcttctg  catgttgtgtggggctgtagtt  aaatgtaccagactccccaaaaa  Tcagtttttagccgggaaggt  caatacgccagcaaaatacctg  Catttgacaggctgatgtctctc  Gctcctcaatccctaactgaagc  Agttcttgcggggaggagtt  Caattctgaacaaacggcattc | |
| **Orientation** | | | | | | | | | |
| C: forward  C: reverse  SSRT C: forward  SSRT C: reverse  D: forward  D: reverse  SSRTD: forward  SSRT D: reverse  F: forward  F: reverse  SSRT F: forward  SSRT F: reverse | | | JH410  JH411  JH513  JH514  JH248  JH249  JH400  JH401  JH583  JH584  JH745  JH765 | | | | Cgattgatttcctactacttcagga  gcctgtttatttgtccccaaga  Cctttgtccatgtggtcaagtt  gagctgatttttaaaaggctagtgct  cgacacatggtccaatcactgtt  agactcccctaggactgccatct  catcaggtcccttctaccatcc  ctaagtctccgcggttgttttc  Acgcatttatttattttgcaacagctgc  Gctgggagaggttcattgaaaacaaca acgcatttatttattttgcaacagc  agaggttcattgaaaacaacaacag | | |
| **qRT-PCR and RT-PCR expression** | | | | | | | | | |
| *IGF1R* : forward  *IGF1R* : reverse  *IRAIN* : forward  *IRAIN* : reverse  *ACTIN*: forward  *ACTIN*: reverse | | | | JH217  JH218  JH248  JH249  J880  J881 | | | gaagtctggctccggaggagggtc  Atgtggaggtagccctcgatcac  cgacacatggtccaatcactgtt  agactcccctaggactgccatct  agatcaagatcattgctcctcctga  atactcctgcttgctgatccacatc | | |
| **Imprinting expression** | | | | | | | | | |
| D’: forward  D’: reverse  E: forward  E: reverse  K: forward  K: reverse  M: forward  M: reverse  Sequencing: forward | | | | | JH248  JH401  JH780  JH781  JH864  JH865  JH891  JH892  JH248 | | | | cgacacatggtccaatcactgtt  ctaagtctccgcggttgttttc  gtttccgcagtagccgctgat  Ctgcgggtctccgaagctc  Ccttggaagatggaagaccgtgt  ggtgccagagaatagttggctcat  atgagccaactattctctggcacc  accctggaaggagccaacgtga  cgacacatggtccaatcactgtt |
| **DNA methylation** | | | | | | | | | |
| Forward  Reverse | JH852  JH855 | | | | | Tygggggatggaggggtattagggt （y=c/t）  cccracaccaccaaaacaaaaActatc（r=g/a） | | | |
| **RAT** | | | | | | | | | |
| a: forward  a: reverse  y: forward  y: reverse  h2: forward  h2: reverse  D’: forward  D’: reverse  E: forward  E: reverse  a1: forward  a1: reverse  a2: forward  a2: reverse  j: forward  j: reverse  k: forward  k: reverse  l: forward  l: reverse  p: forward  p: reverse  u: forward  u: reverse  x: forward  x: reverse  a6: forward  a6: reverse  a7: forward  a7: reverse  input:forward  input: reverse | JH333  JH334  JH377  JH378  JH946  JH947  JH248  JH401  JH780  JH781  JH904  JH906  JH909  JH910  JH485  JH486  jh489  jh490  JH463  JH464  JH225  JH226  JH383  JH384  JH339  JH340  JH864  JH865  JH866  JH870  JH507  JH508 | | | | | gcagaaaatagaagctgcaatcc  tgctgctgcaggtctattacaaa  ttaacctgaggcccaagtatgg  Cacttcaaacagcaatttttcacc  cgacacatggtccaatcactgtt  agactcccctaggactgccatct  cgacacatggtccaatcactgtt  ctaagtctccgcggttgttttc  gtttccgcagtagccgctgat  Ctgcgggtctccgaagctc  ctag gcgcgaggac tcggta  Acgcggtgcggcccagggtg  cctcccgtcgcccaacgtgcttttgccActcgg  caccgcccagccccgGgacgccggTggcaggta  Tcagtttttagccgggaaggt  caatacgccagcaaaatacctg  Agttcttgcggggaggagtt  Caattctgaacaaacggcattc  Tgccgagggtacctggtcttag  Ttctgctctaacacctccagattc  ctgctgtggacttgagtctt  ccaagctgagagcattgctaagg  ttcccttccctgctagtacctg  Ctgcatttctttgcaggtttcc  ccaatggaaggaacagacgttta  Ccattgtggtcagaaaagatacttg  Ccttggaagatggaagaccgtgt  ggtgccagagaatagttggctcat  Ctggccttgcccgtcacttt  tgaaaccacagCGtaCGacacctctCG  Tgtgatcaggttgaaaataacttg  ttgtggtcaatgtggcatatca | | | |
| **3C primers** | | | | | | | | | |
| d’  d’ nested  h1’  h1’ nested  h2’  h2’ nested  j’  j’ nested  p’  p’ nested  s’  s’ nested  x’  x’ nested | | | | | JH738  JH739  JH742  JH743  JH764  JH765  JH740  JH741  JH766  JH767  JH768  JH769  JH756  JH757 | | | | Agccttcagacttggttacccct  AgccttcagacttggttacccctAGT  gggctagacttgctggatcgtaa  Gggctagacttgctggatcgtaagtt  Gctgggagaggttcattgaaaaca  Gctgggagaggttcattgaaaacaaca  gcaagacggcgatttttgagaac  Gcaagacggcgatttttgagaacaca  caGtgaaagggatttgaggcaaaa  cagtgaaagggatttgaggcaaaagat  tgacatgccttacaaacgggtagac  Tgacatgccttacaaacgggtagaccaa  tgtgcaaaacaaaatggcagttc  tgtgcaaaacaaaatggcagttcctt |
| **shRNA** | | | | | | | | | |
| shIRAIN1  shIRAIN2  shCT | | | | |  | | | | GAGCGAAGACTGAGTTTGA  TGGCCGACGAGTGGAGAAA  TTCTCCGAACGTGTCACGT |
| **ChIP** | | | | | | | | | |
| A: forward  A: reverse  B: forward  B: reverse  h2’ :forward  h2’ : reverse  i:forward  i: reverse  j:forward  j: reverse  k: forward  k: reverse  p’ : forward  p’ : reverse  u: forward  u: reverse  x: forward  x: reverse | | | | | JH333  JH334  JH375  JH376  JH248  JH249  JH246  JH247  JH485  JH486  JH489  JH490  JH225  JH226  JH383  JH384  JH339  JH340 | | | | gcagaaaatagaagctgcaatcc  tgctgctgcaggtctattacaaa  cagagcccatcctaaaatcacc  Gggcagtgtttctctgaatgtg  cgacacatggtccaatcactgtt  agactcccctaggactgccatct  ggga gtcttcctca gcttgtctc  acacccaccaacgcacactc  Tcagtttttagccgggaaggt  caatacgccagcaaaatacctg  Agttcttgcggggaggagtt  Caattctgaacaaacggcattc  ctgctgtggacttgagtctt  ccaagctgagagcattgctaagg  ttcccttccctgctagtacctg  Ctgcatttctttgcaggtttcc  ccaatggaaggaacagacgttta  Ccattgtggtcagaaaagatacttg |
| chromatin bound RNA assay | | | | | | | | | |
| 1：forward  1：reverse  2：forward  2：reverse  3：forward  3：reverse  4：forward  4：reverse | | | | | JH217  JH218  JH248  JH249  JH234  JH235  JH387  JH388 | | | | gaagtctggctccggaggagggtc  Atgtggaggtagccctcgatcac  cgacacatggtccaatcactgtt  agactcccctaggactgccatct  gggtgcaccgttcctggaggtac  tcttatcagtttaatatctgatacgtc  Accctcttgtctccctt cagtc  GAAGCGGTAGCTGCGGTAGT |

The optimal 65°C PCR annealing temperature was used for PCR primers listed above.

**ReferenceS**

1. Mondal, T., Rasmussen, M., Pandey, G.K., Isaksson, A. and Kanduri, C. (2010) Characterization of the RNA content of chromatin. *Genome Res*, **20**, 899-907.
